# Structure-function analysis of the DNA-binding domain of a transmembrane transcriptional activator

**DOI:** 10.1038/s41598-017-01031-9

**Published:** 2017-04-21

**Authors:** Andreas Schlundt, Sophie Buchner, Robert Janowski, Thomas Heydenreich, Ralf Heermann, Jürgen Lassak, Arie Geerlof, Ralf Stehle, Dierk Niessing, Kirsten Jung, Michael Sattler

**Affiliations:** 1 Munich Center for Integrated Protein Science (CiPSM) at the Department of Chemistry, Technische Universität München, 85748 Garching, Germany; 2 Institute of Structural Biology, Helmholtz Zentrum München, 85764 Neuherberg, Germany; 3 Munich Center for Integrated Protein Science (CiPSM) at the Department of Microbiology, Ludwig-Maximilians-Universität München, 82152 Martinsried, Germany; 4 Group Intracellular Transport and RNA Biology at the Institute of Structural Biology, Helmholtz Zentrum München, 85764 Neuherberg, Germany; 5 Department of Cell Biology at the Biomedical Center, Ludwig-Maximilians-Universität München, 82152 Martinsried, Germany

## Abstract

The transmembrane DNA-binding protein CadC of *E. coli*, a representative of the ToxR-like receptor family, combines input and effector domains for signal sensing and transcriptional activation, respectively, in a single protein, thus representing one of the simplest signalling systems. At acidic pH in a lysine-rich environment, CadC activates the transcription of the *cadBA* operon through recruitment of the RNA polymerase (RNAP) to the two *cadBA* promoter sites, Cad1 and Cad2, which are directly bound by CadC. However, the molecular details for its interaction with DNA have remained elusive. Here, we present the crystal structure of the CadC DNA-binding domain (DBD) and show that it adopts a winged helix-turn-helix fold. The interaction with the *cadBA* promoter site Cad1 is studied by using nuclear magnetic resonance (NMR) spectroscopy, biophysical methods and functional assays and reveals a preference for AT-rich regions. By mutational analysis we identify amino acids within the CadC DBD that are crucial for DNA-binding and functional activity. Experimentally derived structural models of the CadC-DNA complex indicate that the CadC DBD employs mainly non-sequence-specific over a few specific contacts. Our data provide molecular insights into the CadC-DNA interaction and suggest how CadC dimerization may provide high-affinity binding to the Cad1 promoter.

## Introduction

**Figure 1:**
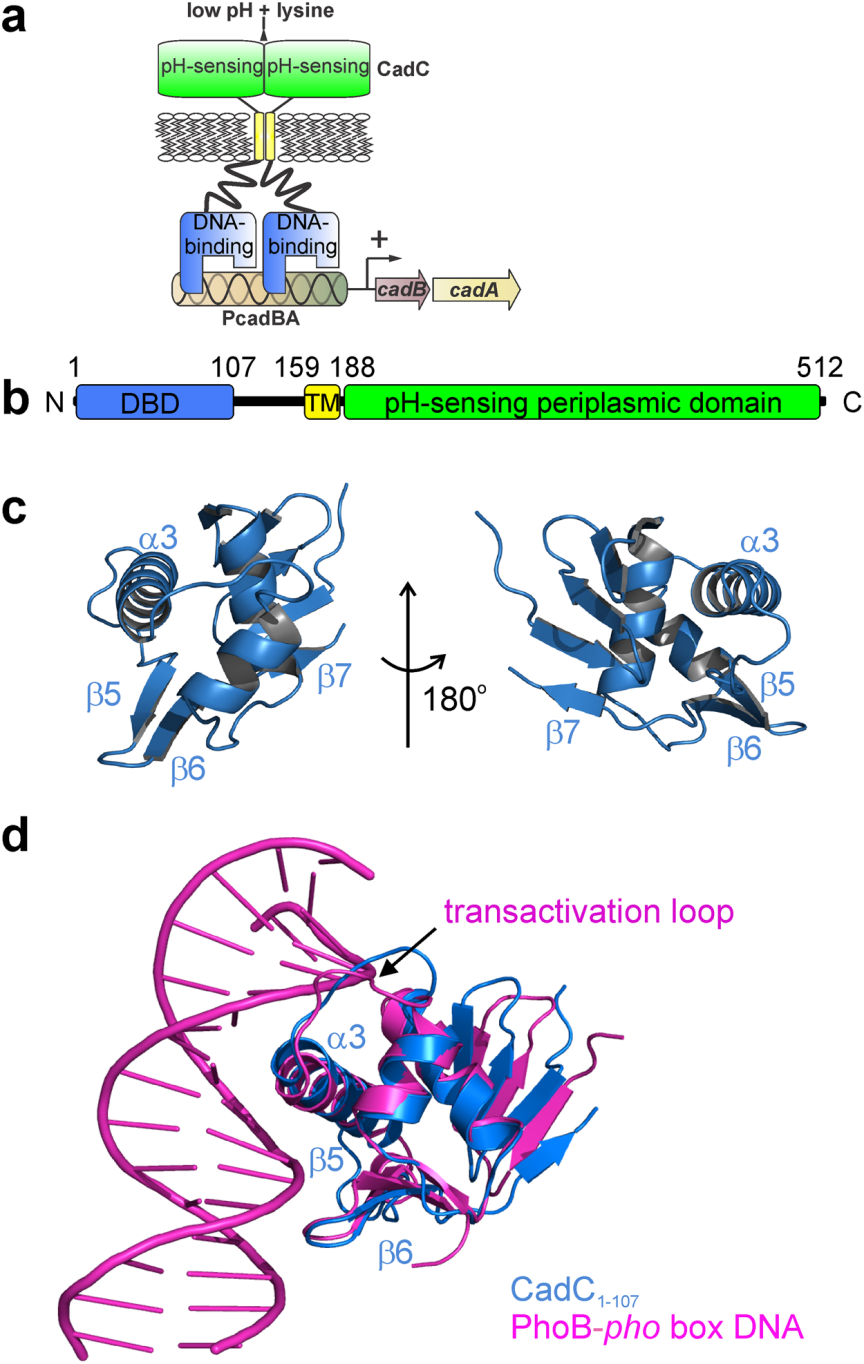
Crystal structure of the CadC DNA-binding domain (DBD). (**a**) Scheme showing the transcriptional activation of *cadBA* genes by CadC in dependence of low pH and increased lysine concentration. The mechanism requires dimerization and therefore two DBDs are shown to bind the *cadBA* promoter (PcadBA). (**b**) Scheme showing the domain organization of CadC from *E. coli*. The DNA-binding domain is shown in blue. Numbers indicate amino acid positions. TM, transmembrane domain. (**c**) Cartoon representation of the *E*. *coli* CadC_1-107_ DBD from two views. Selected secondary structure elements are annotated. (**d**) Superposition of CadC_1-107_ (blue) as in (**c**) with the bacterial transcriptional activator PhoB in complex with DNA (magenta)^16^. Note that only one PhoB monomer and only part of the DNA are shown for clarity. Secondary structure elements involved in DNA-binding by CadC are annotated. The PhoB transactivation loop necessary for binding to RNA polymerase is indicated. Pictures were created with PyMol (Delano Scientific).

Neutrophilic enterobacteria, such as *Escherichia coli*, *Salmonella typhimurium*, and *Vibrio cholerae*, possess different stress response systems to counteract a decrease of external pH by adjusting the pH to a physiological range. One system used by *E. coli* is the Cad-system^1^, that consists of the cytosolic protein CadA and the membrane proteins CadB and CadC (Fig. 1a). The lysine decarboxylase CadA converts lysine into cadaverine and CO_2_ under consumption of one proton. The antiporter CadB transports the basic product cadaverine in exchange for lysine out of the cell^2, 3^. The pH-receptor CadC senses a drop of the external pH and activates transcription of *cadBA* below pH 6.6^4, 5^. This process requires the simultaneous presence of lysine, which is sensed by the co-sensor LysP, a lysine-specific transporter^6, 7^.

CadC belongs to the family of ToxR-like receptors. This protein family is named after ToxR, a membrane-bound transcriptional regulator that controls virulence in *V. cholerae* ^8^. Other members of this protein family are TcpP, TfoS, and VttR_A_ and VttR_B_ in *V. cholerae* ^9–12^, PsaE in *Yersinia pseudotuberculosis* ^13^, WmpR in *Pseudoalteromonas tunicata* ^14^, and ArnR in *Sulfolobus acidocaldarius* ^15^. ToxR-like receptors are characterized by a conserved modular composition: external signals are sensed by a periplasmic sensor domain and are transduced through a single transmembrane helix to a cytoplasmic DBD of an OmpR type, which acts as effector domain to regulate gene expression. Hence, signal transduction appears to be independent of chemical modifications like phosphorylation. Despite their simple design principle, the exact mechanism of signal transduction has remained enigmatic for this receptor family. Mechanisms of transcription enhancement by OmpR-type effector domains of transcriptional activators have been studied biochemically for many bacterial response systems. However, structural information of these effector domains in complex with target DNA is limited to a few examples^16–18^ and no ToxR-like DBD structures are available to date.

Functional activity depends on CadC dimerization^5, 19^ (Fig. 1a). The structure of the periplasmic sensory domain of CadC and molecular features of the pH-sensing mechanism have been described previously^5, 20^. A patch of negatively charged amino acids located at the dimer interface is involved in pH sensing^5^. Protonation of these amino acids might reduce repulsing forces between the two monomers and thereby enable receptor activation. To investigate how the signal is processed in the cytoplasm, we have recently studied the role of the linker that connects the transmembrane helix and the DBD. We found that the linker is intrinsically disordered, but nevertheless required for proper positioning of the two DBDs for activation of transcription^21^. CadC-mediated stress response requires binding of the protein to two DNA-bindings sites, Cad1 and Cad2 within the *cadBA* promoter region^22^. It has been proposed that CadC targets a quasi-palindromic sequence within Cad1^22^. However, the exact binding sites have not been mapped.

Here, we investigate the structural basis for DNA recognition by the CadC DBD. We present the crystal structure of the CadC DBD and analyse its interaction with DNA using NMR spectroscopy and complementary biophysical and functional techniques. We define the binding stoichiometry and affinities with the Cad1 DNA promoter and regions within. Mutational analysis identified amino acids in the CadC DBD that are important for DNA recognition and function. Analysis of the interaction of the CadC DBD with DNA demonstrates that CadC mainly utilizes non-sequence-specific and only few specific interactions for complex formation. NMR and *in vitro* binding data indicate a preference of CadC for binding to AT-rich regions within the Cad1 promoter. Finally, a structural model of the CadC DBD-DNA complex based on our experimental data provides a molecular rationale for the distinct binding preferences of the CadC and other DBDs found in bacterial response regulators.

## Results

### Crystal structure of the CadC DNA-binding domain

To characterize the CadC effector domain and the interaction with DNA, we first determined the crystal structure of the CadC DBD (amino acids 1–107, Fig. 1b and c) at 2.0 Å resolution (Supplementary Table S1 and Supplementary Fig. 1). Consistent with our recent study of the complete cytoplasmic part of CadC^21^ small angle X-ray scattering (SAXS) data indicate that the DBD is a monomer in solution (Supplementary Table S2). The overall fold and secondary structure is characteristic for the winged helix-turn-helix (HTH) motif found in members of the OmpR family effector domains. Thus, CadC comprises a recognition helix (α3) and a β-hairpin wing (β5-loop-β6) within a compact fold with the topology β1-β2-β3-β4-α1-α2-α3-β5-β6-β7. To our knowledge, the CadC DBD represents the first structure of a ToxR-like DBD.

We performed structural similarity searches using DALI^23^ (Supplementary Fig. S1). Comparing CadC_1-107_ with the eleven best-scored hits revealed overall highly similar folds. A unique feature of the CadC DBD is an additional short β-strand (β7) that extends the highly conserved C-terminal β-sheet (β1-β4) in OmpR-type DBDs. Consequently, the β-sheet in CadC is shifted towards the site of the β4 strand to maintain a compact globular fold. To rule out that these conformational differences reflect crystal-packing artefacts we analysed the CadC DBD in solution using heteronuclear NMR spectroscopy. Secondary ^13^C chemical shifts (Supplementary Fig. S1) show that the additional C-terminal β7-strand (residues 104–106) is also present in solution. This is confirmed by ^15^N, and ^13^C-edited nuclear Overhauser effect spectroscopy (NOESY) data, which show specific contacts between strands β1 and β7 (Supplementary Fig. S1). The additional strand β7 is important for the structural integrity of CadC, as a truncated variant (CadC_1-102_) was less soluble and unstable. Nevertheless, a CadC variant with the three amino acids V104, I105 and W106 replaced by alanine, supports stress-dependent signalling like wild-type CadC (data not shown). This implies that the integrity of the β7 strand is not critical for signal transduction. The highest structural similarity of CadC_1-107_ with an effector domain bound to DNA is found with PhoB. The structure of the PhoB DBD in complex with *pho* box DNA^16^ superimposes with the CadC DBD with a backbone coordinate RMSD (root-mean-square deviation) of 1.8 Å (Fig. 1d). Given the structural similarity, the CadC-DNA binding interface is expected to resemble the PhoB-DNA interaction.

### DNA-binding of CadC to the *cadBA* promoter

**Figure 2:**
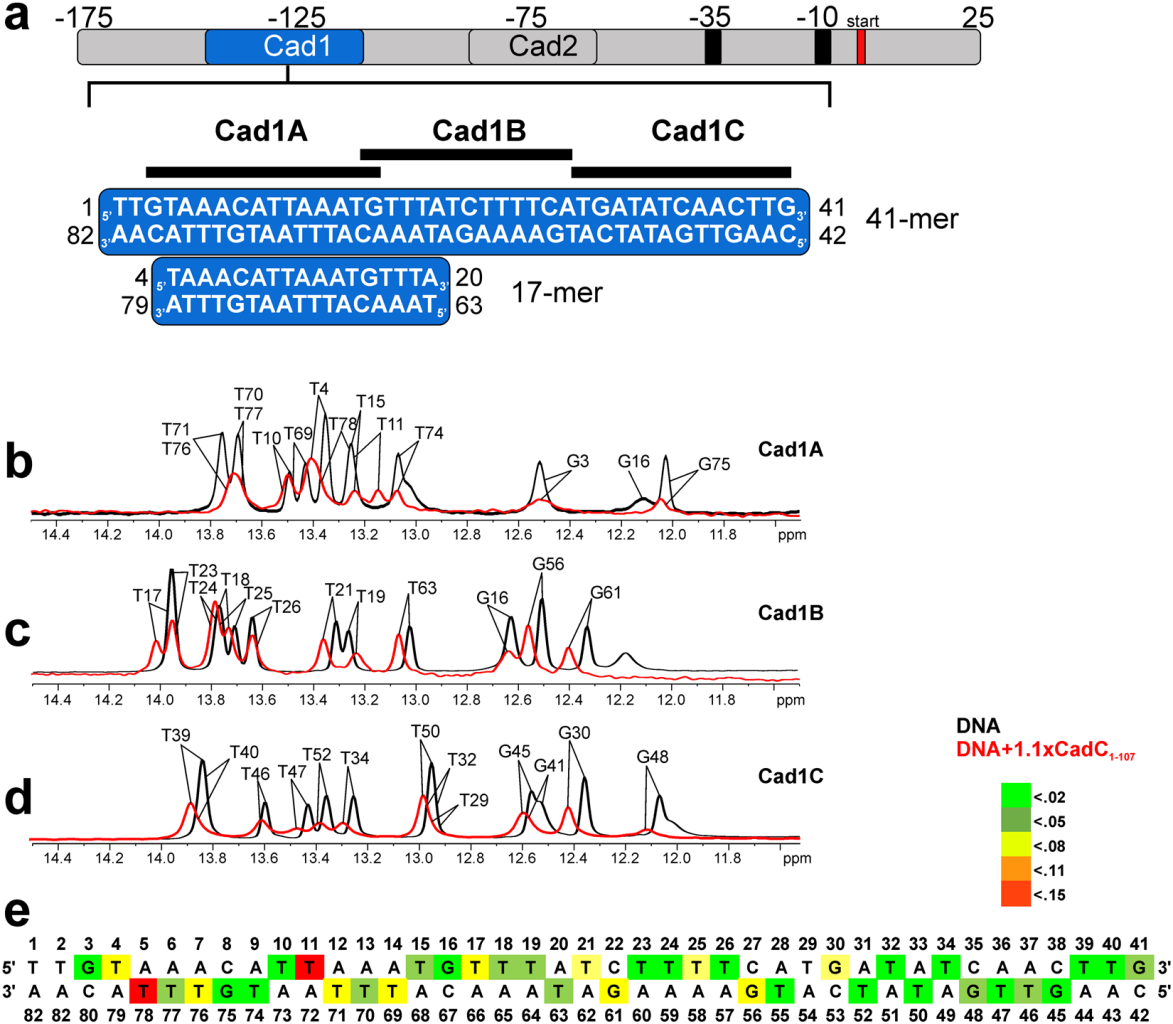
NMR-based analysis of Cad1 sub-fragment binding to CadC_1-107_. (**a**) Top, scheme of the full *cadBA* promoter region between −175 bp and 25 bp relative to the transcription start with CadC-binding sites Cad1 and Cad2 and the RNA polymerase binding sites at −35 bp and −10 bp. Bottom, scheme of fragments Cad1A, B and C within the Cad1 41-mer sequence used in this study. Numbers represent the internal numbering along full-length Cad1 with the boundaries given. The previously suggested CadC target sequence^22^ (Cad1 17-mer) is﻿﻿ shown for comparison (see Supplementary Fig. S4). (**b**–**d**) Imino proton spectra of fragments Cad1A, B and C alone (black) or in stoichiometric excess of CadC_1-107_ (red). Assignments are indicated. (**e**) Quantification of chemical shift perturbations (CSPs) from panels (**b**–**d**). The colour indicates categories of CSP effects, i.e. CS differences.

CadC binding to the *cadBA* DNA promoter site depends on the complete Cad1 region^22^ (Fig. 2a and Supplementary Fig. S2). By comparing NMR spectra of the CadC DBD alone (CadC_1-107_) with a construct comprising the DBD and the cytoplasmic linker (CadC_1-159_), we have recently shown that amino acids within the flexible linker do not interact with the DBD and DNA^21^. We then defined the DNA-binding surface in the CadC DBD by following NMR chemical shift changes in ^1^H,^15^N correlation experiments upon titration of a double-stranded DNA oligonucleotide encompassing the Cad1 binding site (−153 to −113 bp) (Cad1 41-mer, Supplementary Fig. S2)^22^. Amide signals in CadC_1-159_ were monitored after titration of 41-mer DNA to a final protein:DNA ratio of 2:1. The spectrum shows severe line broadening, which reflects a significant increase in molecular weight upon complex formation and/or dynamics due to sliding of the protein on the DNA (Supplementary Fig. S2). The 2:1 complex was subsequently confirmed in static light scattering (SLS) and SAXS measurements (Supplementary Fig. S2). NMR signals that remain observable are located in loops of the DBD and the flexible CadC linker beyond residue 107. No chemical shift perturbations (CSPs) are observed for these residues indicating that these residues are not involved in DNA-binding.

### The role of AT-rich patches in Cad1

Previous studies^22, 24^ suggested that CadC preferentially binds to AT-rich sequence stretches of DNA. AT-rich regions of a DNA duplex exhibit a narrowing of the minor groove and thus may increase the contribution of specific contacts to the overall affinity^25^. To examine the *cadBA* promoter and map CadC binding sites in the Cad1 41-mer we studied three smaller regions, Cad1A (14-mer), Cad1B and Cad1C (13-mers) (Fig. 2a), all of which are of a length that is typically covered by DBDs^16, 18^. Notably, Cad1A and Cad1B together harbour a Cad1 17-mer sequence, which had been previously suggested as CadC target site ^22^. While the Cad1A and Cad1B DNA sequences are highly similar and exhibit a high AT-content with almost inverse/head-to-head sequence motifs, Cad1C lacks significant AT-patches. ^1^H NMR spectra of these DNAs with and without CadC show that all three DNAs bind to the protein (Fig. 2b–d). We assigned the imino proton NMR chemical shifts in both the DNA fragments alone and when in complex with CadC_1-107_ and analysed the effects of binding (Fig. 2e). An analysis of the CSPs reveals that CadC causes stronger effects upon binding to Cad1A and Cad1B than to Cad1C, indicating a preference of the CadC DBD for binding to AT-rich DNA.

**Figure 3:**
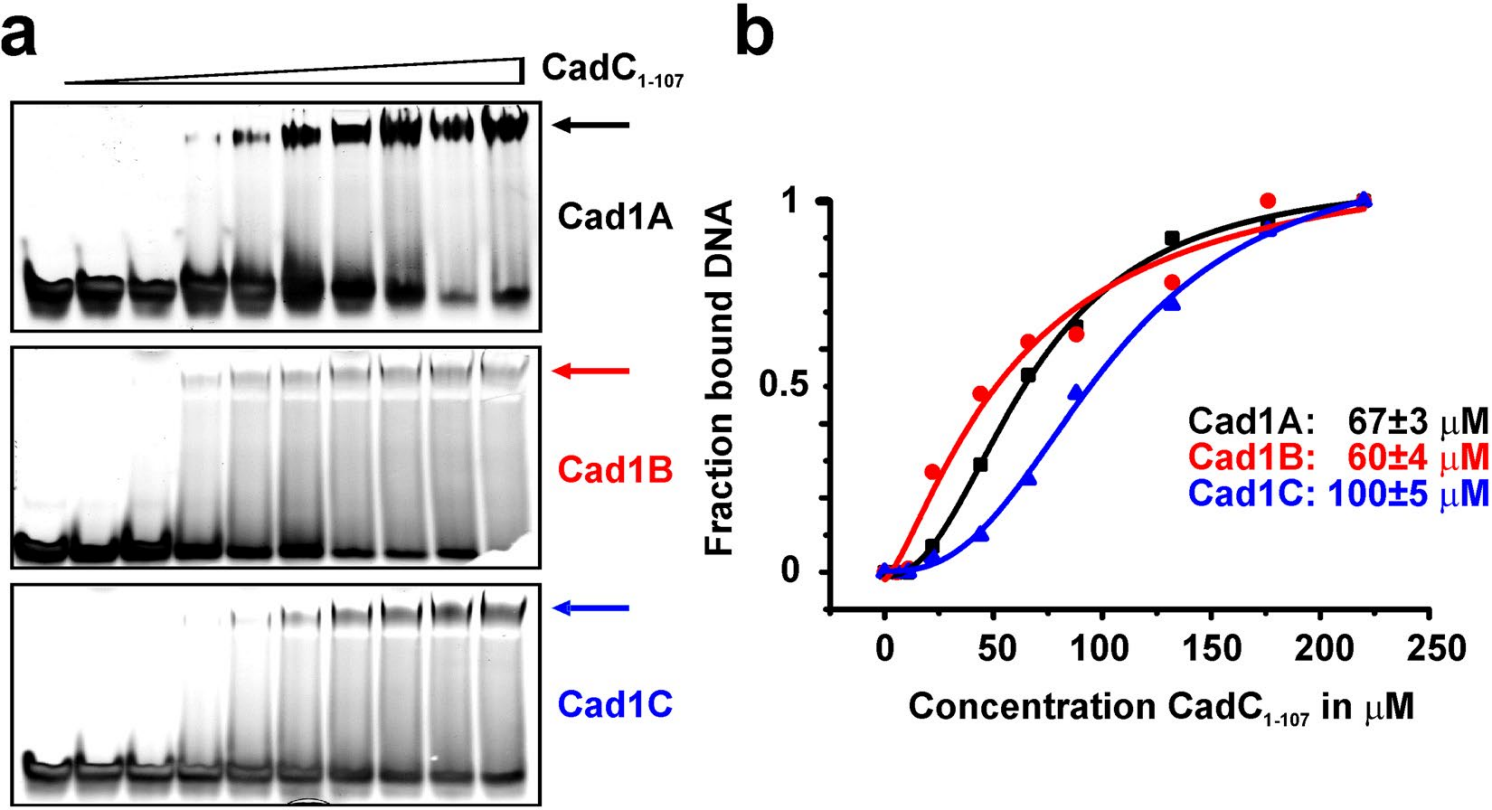
EMSA-based analysis of Cad1 fragments. (**a**) Representative EMSA analysis of fragments Cad1A, B and C when titrated with CadC_1-107_ to ten-fold stoichiometric excess. Arrows indicate the complex species. (**b**) Quantification of EMSA experiments in (**a**). Binding constants are given as obtained from a one-site binding event and shown as mean ± standard deviation.

We next quantified binding affinities of CadC to the three fragments using electromobility shift assays (EMSAs) (Fig. 3a and b). The two AT-rich fragments Cad1A and Cad1B show similar affinities with low micromolar dissociation constants while Cad1C is bound with a slightly lower affinity. These values are consistent with the binding of other DBDs to promoter half-sites^17, 26^. We then monitored the binding of these DNA regions to CadC in ^1^H,^15^N correlation experiments with ^15^N-labeled CadC_1-107_ (Supplementary Fig. S3). All NMR spectra show significant spectral changes, indicating binding of all three DNAs to CadC. Interestingly, line-broadening is observed for residues in the protein-DNA interface upon titration of Cad1C. In contrast, little line-broadening is observed during the titration of the AT-rich Cad1A and Cad1B DNA duplexes. This indicates that the interaction of Cad1C exhibits dynamics at μs-ms time scales. Possibly, the lack of AT-rich regions in Cad1C leads to sliding and is consistent with a reduced binding affinity to CadC compared to Cad1A and Cad1B. These data show that CadC recognizes shorter fragments in the Cad1 promoter with a preference for AT-rich regions.

**Figure 4:**
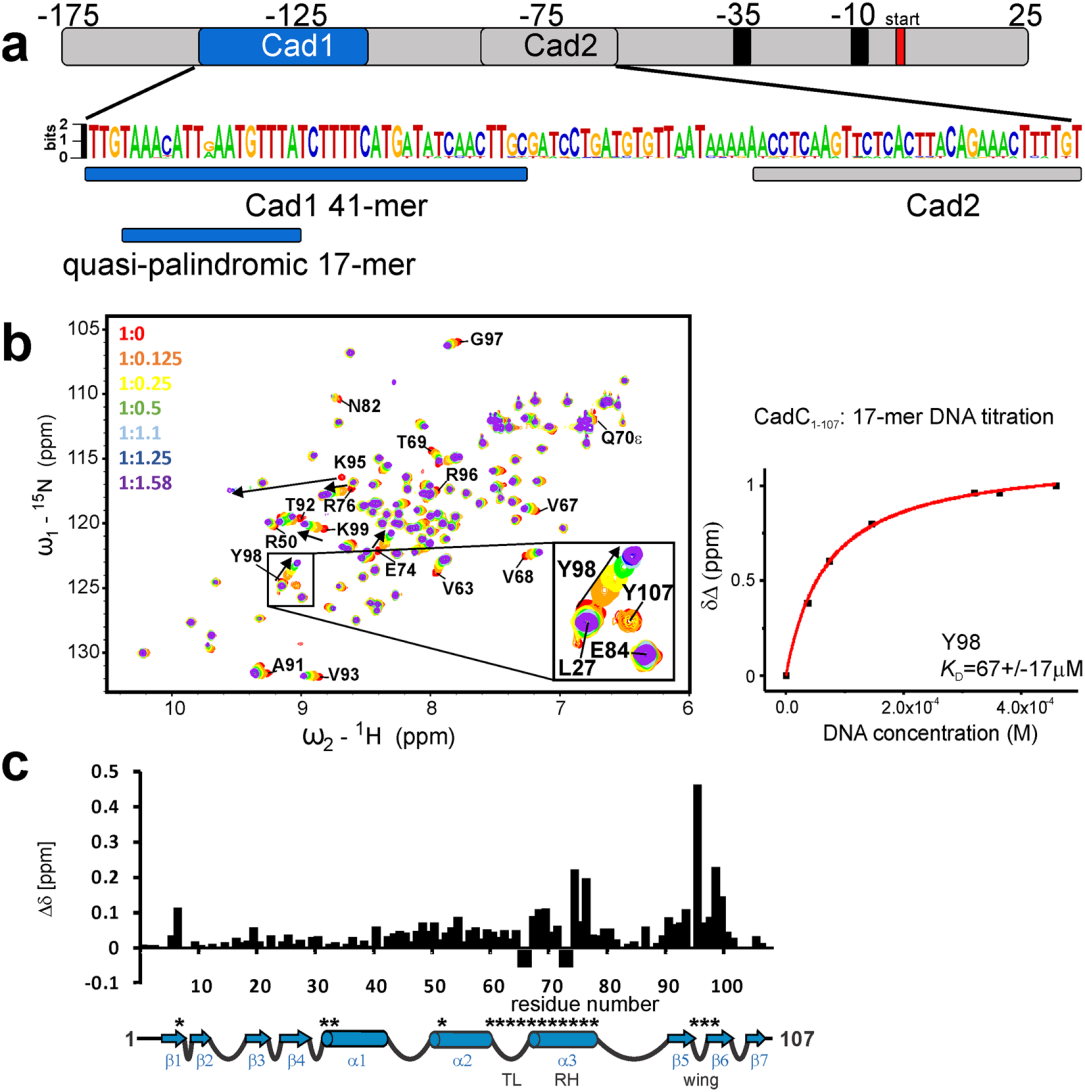
NMR analysis of CadC_1-107_ binding to Cad1 DNA. (**a**) Top, the scheme shows the full *cadBA* promoter region between −175 bp and 25 bp relative to the transcription start with CadC binding sites Cad1 and Cad2, and the RNA polymerase binding sites at −35 bp and −10 bp. Middle, web-logo showing the conservation of nucleotides in the Cad1 and Cad2 fragments of the *cadBA* promoter region for enteric bacteria according to NCBI blastn^75^. The loci of the Cad1 and Cad2 sites are indicated. Blue bars represent the Cad1 41-mer and the quasi-palindromic 17-mer core sequences used in this study. (**b**) Left, superposition of ^1^H-^15^N-HSQC spectra from CadC_1-107_ in the absence or presence of different amounts of Cad1 17-mer DNA (see colour codes) with selected residues depicted. The inset shows a magnification of selected amide resonances. Right, quantification of CSPs of Tyr 98 (see inset) by fitting relative CSPs to a 1:1 binding model. The given binding constant is calculated from all significantly shifting resonances and shown as mean ± standard deviation. (**c**) Plot of CSPs, i.e. the difference of chemical shifts of free CadC and the complex with maximum DNA (1:1.58). Gaps indicate prolines; negative bars are residues in which the assignment in either the bound or apo-form is missing. The scheme at the bottom of the diagram shows the secondary structure of the CadC DBD based on the crystal structure with elements annotated. Asterisks indicate positions that were used for mutational analyses at a later stage of the study. TL, transactivation loop; RH, recognition helix; wing, β-wing.

Interestingly, the Cad1A and Cad1B DNAs represent an AT-rich quasi-palindromic 17-mer motif (Figs 2a and 4a) which has been previously suggested to be the CadC interaction site within Cad1^22^. We therefore studied binding of CadC_1-107_ to this Cad1 17-mer in NMR titrations. Spectral changes observed during the titration show binding kinetics in fast-exchange on the NMR chemical shift time scale^27^ (Fig. 4b), typically reflecting micromolar affinities (*K*_D_). Quantitative analysis of the 17-mer NMR titration data yields a *K*_D_ = 67 ± 16 μM for the interaction (Fig. 4b, right panel), consistent with surface plasmon resonance data that indicate a steady state constant *K*_D_ = 23 μM (Supplementary Fig. S4).

Based on the NMR spectral changes the binding interface with the 17-mer DNA (Fig. 4c) maps to two regions comprising the recognition helix α3 and the stretch between amino acids 90 and 100, i.e. the loop contacting the DNA minor groove in the hypothetical model with *pho*-box DNA (Fig. 1d). To map the binding interface from the side of the DNA we analysed chemical shift changes of the imino protons of the Cad1 17-mer DNA duplex upon addition of the CadC DBD (Supplementary Fig. S4). Interestingly, only thymine imino signals are perturbed upon addition of protein while no guanosine iminos are affected, demonstrating that the CadC DBD preferentially contacts A-T base pairs. This provides further evidence for the preference of CadC in binding to AT-regions within the *cadBA* promoter.

### Stoichiometry of CadC-DNA complexes

Using NMR and SLS measurements we have previously shown that the cytoplasmic portion of CadC is monomeric independent of the concentration^21^. We then characterized the oligomeric state and binding stoichiometries of the CadC-DNA interaction (Supplementary Figs S2 and S4). SLS of the Cad1 41-mer with CadC_1-107_ clearly shows that the protein binds in a 2:1 ratio to the DNA (Supplementary Fig. S2). This is further supported by SAXS experiments (Supplementary Fig. S2; Supplementary Table S2). While SAXS data of CadC_1-107_ alone are in excellent agreement with the crystal structure (χ^2^ of 0.98), analysis of the CadC_1-107_ 41-mer Cad1 DNA complex indicates the presence of two domains bound to the *cadBA* promoter DNA (Supplementary Fig. S2). In contrast, SAXS data demonstrate that the Cad1 17-mer DNA binds to CadC_1-107_ with a 1:1 stoichiometry (Supplementary Table S2). This is further supported by the tumbling correlation time of the protein-DNA complex derived from NMR relaxation data, which are in good agreement with the expected molecular weight of a 1:1 CadC DBD complex with 17-mer Cad1 DNA and indicate that the CadC DBD alone is monomeric (Supplementary Fig. S4). Thus, while the CadC DBD forms a 1:1 complex with the 17-mer harbouring an AT-rich region, the complete Cad1 41-mer DNA can adopt a 2:1 complex *in vitro*. *In vivo* this may allow the binding of two DBDs from a membrane-anchored CadC dimer with the promoter.

### Identification of amino acids important for DNA recognition and function of CadC

**Figure 5:**
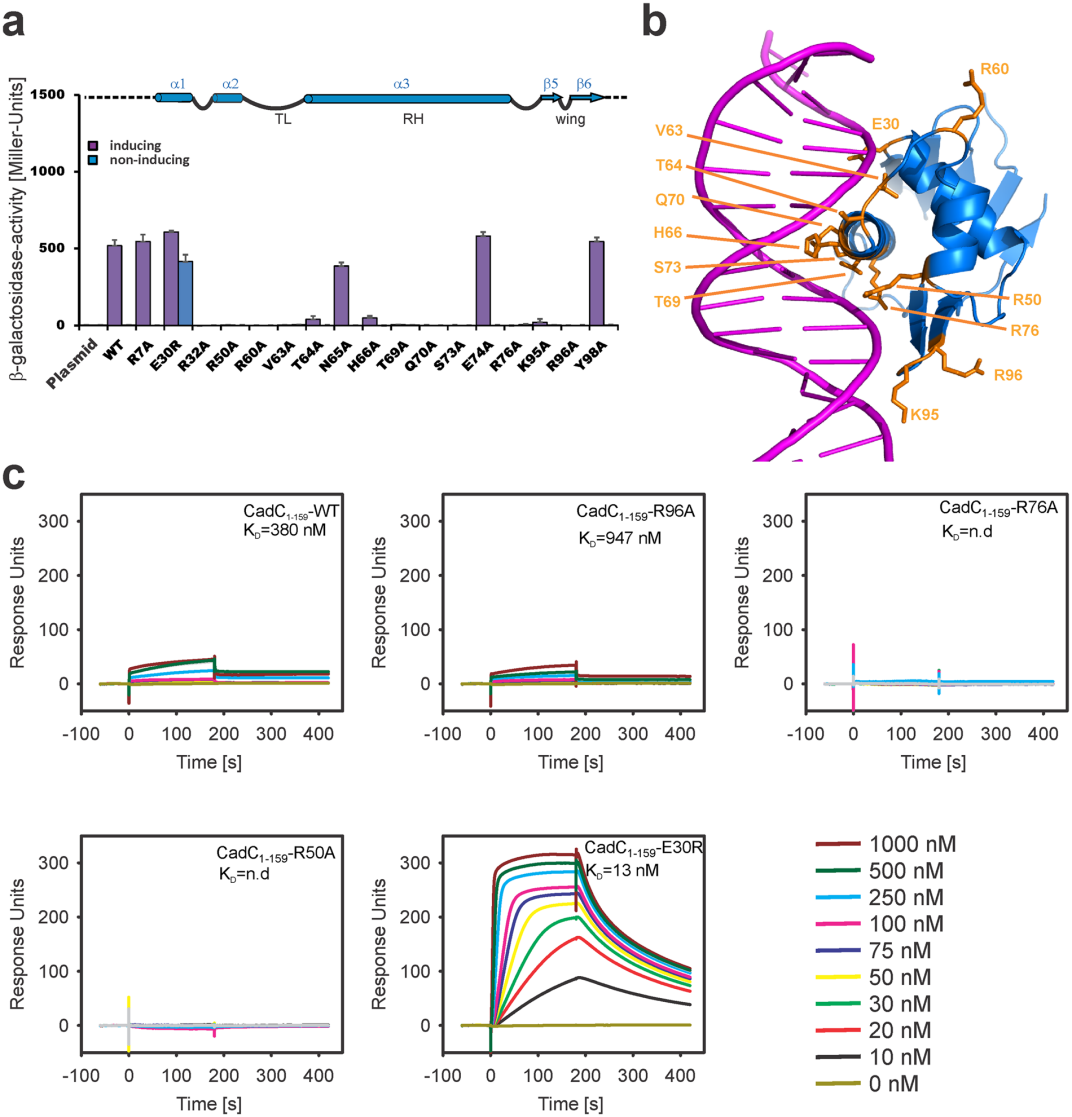
Influence of amino acid substitutions within CadC on the expression of the *cadBA* operon. (**a**) Quantification of *cadBA* transcription in *E. coli* EP314 using the reporter gene *lacZ*. Cells were grown at inducing or non-inducing conditions, and the β-galactosidase activity was determined. The experiment was performed in triplicate, and error bars indicate standard deviations from the means. On top, a part of the CadC secondary structure is shown to give orientation along the elements that are essential for transcriptional regulation. See Supplementary Fig. S5 for all tested experimental conditions. (**b**) Visualization of mutation sites within CadC_1-107_. The structure shows the CadC DBD docked to *pho*-box DNA as in Fig. 1d. Selected side chains from amino acids with significant effect in (**a**) and from CSPs in Fig. 4c are shown as sticks and annotated. (**c**) CadC affinity for Cad1 41-mer DNA. Representative SPR sensorgrams obtained from CadC_1-159_ wild-type (WT) or the respective variants injected in different concentrations (see colour code) to immobilized Cad1 41-mer DNA. Sensorgrams representing real time binding are shown with steady-state dissociation constants. The figures represent each one characteristic of three independently performed experiments. Binding curves to derive the overall steady-state affinities are shown in Supplementary Fig. S5.

The DNA-binding interface suggested by the superimposition with PhoB-*pho* was probed by replacement of selected amino acids in the CadC DBD. These were expected either to be involved in DNA contacts via the recognition helix (RH) and the β-wing or to be important for transcriptional regulation, e.g. through the transactivation loop (TL) (Fig. 1d). To test these CadC variants for *cadBA* promoter activation, we used the *E. coli cadA*::*lacZ* reporter strain EP314^28^. β-Galactosidase activities were determined for cells grown in minimal medium under c*adBA*-inducing (pH 5.8 and lysine) and non-inducing (pH 7.6 without lysine) conditions. We replaced selected charged amino acids and those that were affected in the NMR titration (Fig. 5 and Supplementary Fig. S5). Significantly impaired activity under stress conditions was observed for alanine replacements of Arg32, Arg50, Arg60, Val63, Thr64, His66, Thr69, Gln70, Ser73, Arg76, Lys95 and Arg96 (Fig. 5a). There is an excellent correlation with spatial proximity of the corresponding side chains to the DNA in the structural superimposition of CadC and PhoB that leads to the aligned CadC-*pho*-box DNA complex (Fig. 5b). Notably, these amino acid replacements in CadC abolished activation of the *cadBA* promoter under all tested conditions, demonstrating their functional significance (Supplementary Fig. S5).

In NMR titrations of the CadC DBD with the 17-mer Cad1 DNA, these amino acid replacements strongly affect DNA-binding (Fig. 4b and c). It is noteworthy that the amide NMR signals of Asn65 and His66 could not be assigned in the apo-form of the CadC protein due to line-broadening suggesting that this region is conformationally flexible in the absence of DNA (Fig. 4c and Supplementary Fig. S4). The structural alignment of CadC with PhoB (Figs 1d and 5b) also explains the role of Val63 and Arg60. These two residues are located in the potential TL of CadC and might be involved in interactions with the RNAP. Amino acid replacements in this loop have been previously shown to suppress transcription activation by PhoB^29^.

Surprisingly, the position of Glu30 is extremely sensitive to amino acid substitutions. As Glu30 is expected to be in close proximity to the DNA backbone (Fig. 5b), the introduction of a positive charge (E30R) at this position can enhance the DNA-binding affinity by providing additional contacts to the DNA. This may explain why the E30R mutant induces constitutive *cadBA* promoter activation independent of pH and lysine (Supplementary Fig. S5).

To investigate whether the observed effects with the CadC variants are related to perturbed interactions with DNA we performed SPR (surface plasmon resonance, Biacore) experiments and compared the binding affinity of wild-type (WT) and variant CadC proteins (Fig. 5c). The overall affinity, determined from the steady state binding model, corresponds to an equilibrium dissociation constant *K*_D_ = 380 nM (Supplementary Fig. S5), and is thus 60-fold stronger compared to the Cad1 derived 17-mer DNA (*K*_D_ = 23 μM, Supplementary Fig. S4, see discussion). The R50A and R76A variants almost abolish, while the β-wing variant R96A reduces DNA-binding consistent with the importance of these residues for DNA-binding (Fig. 5a and c). Apparently, the decrease in affinities observed *in vitro* is sufficient to inhibit recruitment and activation of the RNAP on the *cadBA* promoter in cells where hierarchical binding of effector domains and polymerase has been found before^30^. The CadC E30R variant exhibits an almost 30-fold increased affinity (Fig. 5c, Supplementary Fig. S5) consistent with additional DNA contacts involving the arginine side chain.

### Atomic Models of CadC-Cad1 DNA interactions

**Figure 6:**
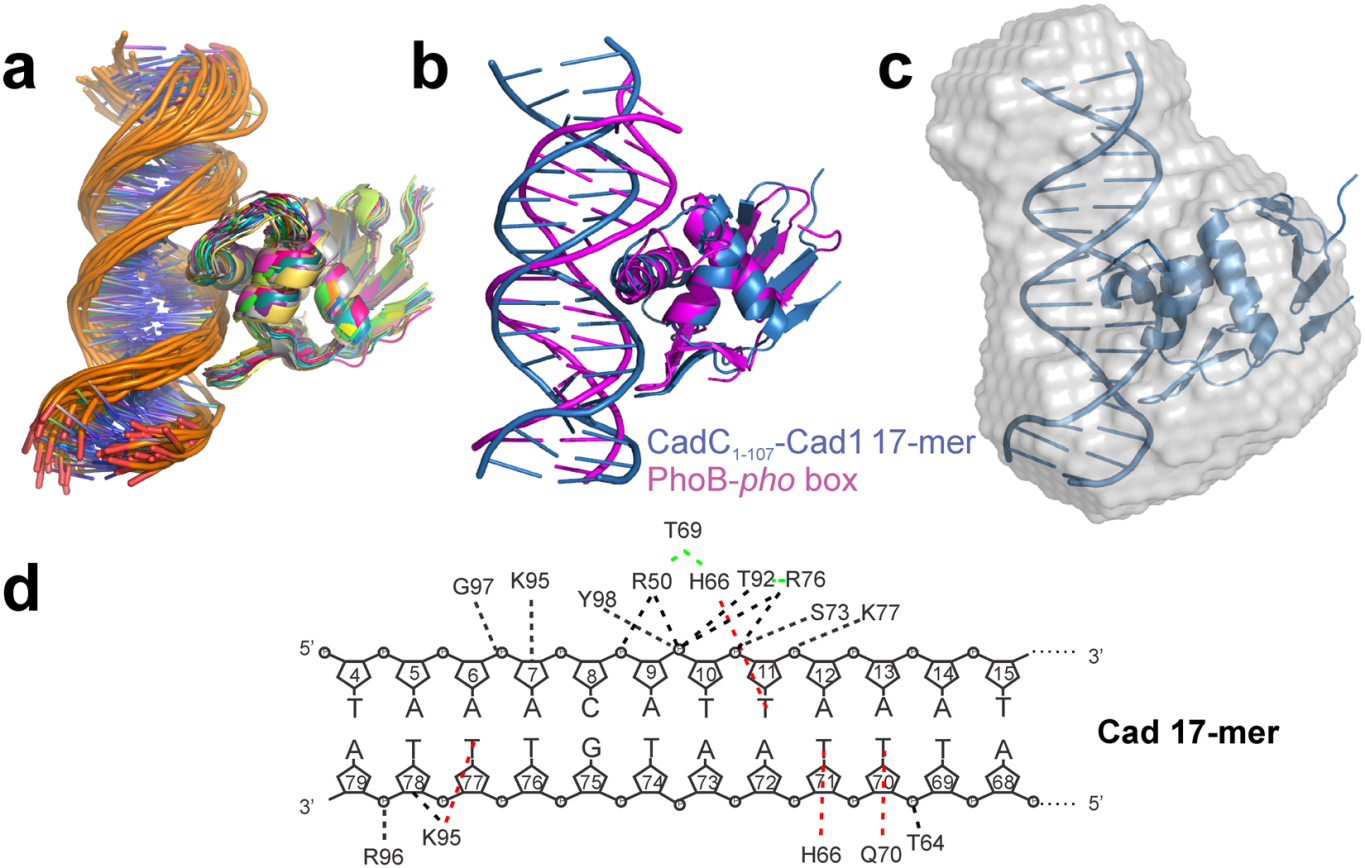
HADDOCK model of the CadC_1-107_ structure with the Cad1 17-mer DNA. (**a**) Overlay of all 26 ensemble structures within the respective cluster from a HADDOCK modelling of CadC_1-107_ with Cad1 17-mer DNA (Supplementary Table S3). (**b**) Alignment of the lowest-energy model for the CadC_1-107_/Cad1 DNA complex from (**a**) with the structure of the PhoB-*pho* box DNA complex (PDB: 1gxp^16^) (see Fig. 1d). DNAs are coloured according to proteins. (**c**) Superposition of a SAXS-derived ab initio shape model of a 1:1 complex of CadC_1-107_ with 17-mer Cad1 DNA (grey, semi-transparent) with the lowest-energy structure from the cluster in (**a**) shown as blue cartoon. (**d**) Contacts of CadC_1-107_ in complex with Cad1 17-mer DNA. Red lines indicate sequence-specific interactions of protein side chains with DNA bases. Black lines are contacts to the DNA backbone, i.e. the phosphate or the sugar groups. Green lines show relevant, sequence-specific protein-protein contacts.

The mutational analysis of key residues in the CadC-DNA complex supports the protein-DNA interactions suggested by the superimposition of CadC with *pho*-box DNA shown in Fig. 5b. To obtain more detailed insight into specific interactions between the CadC DBD and the Cad1 DNA site we generated a docking model based on our experimental information and the structural similarity to PhoB (Figs 1d and 5b) using HADDOCK^31^ (Fig. 6; Supplementary Fig. S6). An overlay of the lowest energy model with the PhoB-DNA complex confirms the overall similarity of the protein-DNA-binding interface (Fig. 6b). A SAXS-derived ab initio model is consistent with the structural model of the CadC_1-107_/17-mer DNA complex (Fig. 6c).

The analysis of the protein-DNA contacts in the docking model (Fig. 6d) highlights the predominant presence of non-sequence specific contacts over only a few sequence-specific interactions. Only the side chains of Lys95, His66 and Gln70 exhibit specific contacts with thymidines at position 77, 11/71, and 70 in the DNA, respectively (Fig. 6d and Supplementary Fig. S6). This is consistent with the importance of the AT-rich sequence in both the 17-mer and the 41-mer DNA ligands. Interestingly, the 12 base pairs in the 17-mer DNA sequence that are recognized by CadC correspond to the Cad1A region, indicating that CadC specifically recognizes a few identical nucleotides in the two DNA fragments. Altogether, the specific contacts observed suggest a consensus binding motif of 5′-T-T-A-x-x-x-x-T-3′. The Cad1 41-mer DNA harbours two of these consensus sites (Supplementary Fig. S6) in a head-to-head arrangement, which is in line with our biophysical analysis and the experimentally observed 2:1 stoichiometry. Depletion of these two sites leads to a reduced affinity of CadC in EMSA and ITC (isothermal titration calorimetry) experiments (Supplementary Fig. S7) consistent with a role for CadC dependent regulation. A more detailed analysis of the docking model also suggests the role of amino acids in CadC that are not directly contacting the DNA (Supplementary Fig. S6), but are still essential for expression of the target operon (Fig. 5). For example, an indirect contribution of Thr69 for DNA-binding is explained by hydrogen-bonds that arrange the His66 and Arg50 side chains, which both are crucial for DNA-binding (Supplementary Fig. S6). Thr69 replacement with alanine resulted in a loss of transcriptional activation of the *cadBA* operon by CadC (Fig. 5 and Supplementary Fig. S5).

**Figure 7:**
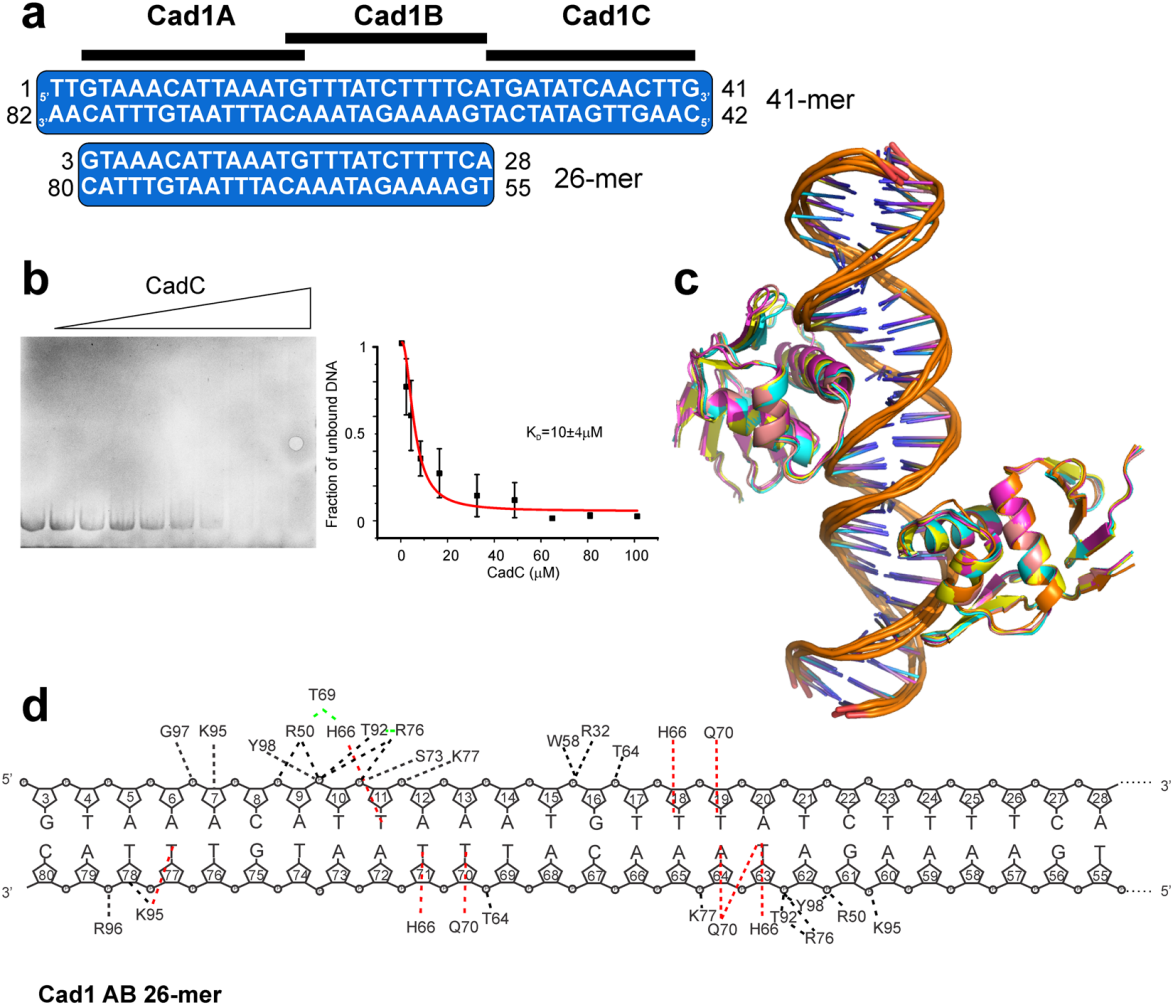
HADDOCK model of the CadC_1-107_ structure with the Cad1AB 26-mer DNA. (**a**) The scheme shows the full Cad1 of the *cadBA* promoter. The new Cad1AB 26-mer spans the sub-fragments A and B. (**b**) Left, shown is a representative gel of Cad1AB 26-mer DNAs from panel (**a**) when titrated up to tenfold excess of CadC_1-107_. The band of free DNA was used for quantification of the complex formation (right). Binding constant is given as obtained from a triplicate dataset and shown as mean ± standard deviation. (**c**) Overlay of all ensemble structures within the respective cluster from a HADDOCK modelling of CadC_1-107_ with Cad1AB 26-mer DNA (see Supplementary Table S3). (**d**) Contacts of CadC_1-107_ in complex with Cad1AB 26-mer DNA. Red lines indicate sequence-specific interactions of protein side chains with DNA bases. Black lines are contacts to the DNA backbone, i.e. the phosphate or the sugar groups. Green lines show relevant, sequence-specific protein-protein contacts.

Based on the presence of a weak double-consensus within the Cad1 41-mer we reasoned that CadC would reveal similar binding propensities for a minimal Cad1 26-mer that comprises the two fragments Cad1A and B (Cad1AB) (Fig. 7a and Supplementary Fig. S8). Indeed, SLS shows a clear 2:1 stoichiometry (Supplementary Fig. S8) and both by EMSA (Fig. 7b) and by ITC (Supplementary Fig. S8) we determined a binding constant identical to the 41-mer. Notably, an HSQC overlay of CadC_1-107_ alone and in complex with Cad1AB shows notable line-broadening (Supplementary Fig. S8). However, the effect is significantly less pronounced than in the NMR titration with Cad1 41-mer DNA. We conclude that the overall CadC_1-107_ binding affinity and stoichiometry to Cad1 41- and 26-mers is comparable but as the 26-mer DNA leaves less space for sliding we observe a more rigid complex as suggested by improved NMR spectral quality. We next aimed for defining imino proton CSPs of Cad1AB in order to use them as restraints in model building of CadC-Cad1AB (Supplementary Fig. S8). However, while the majority of NMR signals could be assigned in the free DNA, strong signal overlap (as a result of the high AT content) prohibits assignment of imino protons in the bound form. We used the protein CSPs to prepare a HADDOCK model using the same approach as described for the 17-mer complex, which yields a head-to-head arrangement of the two DBDs (Fig. 7c). This model is consistent with all available experimental data and also represented by a SAXS-derived overall shape (Supplementary Fig. S8). Interestingly, it does not indicate a direct interaction of the two DBDs, consistent with the lack of strongly enhanced DNA binding affinity compared to the 1:1 complex. Notably, the 2:1 model provides additional contacts, e.g. for Arg32 and Trp58 of CadC with the DNA that well resemble the mutational and NMR CS data and have not been observed in the model with 17-mer DNA (Fig. 7d).

**Figure 8:**
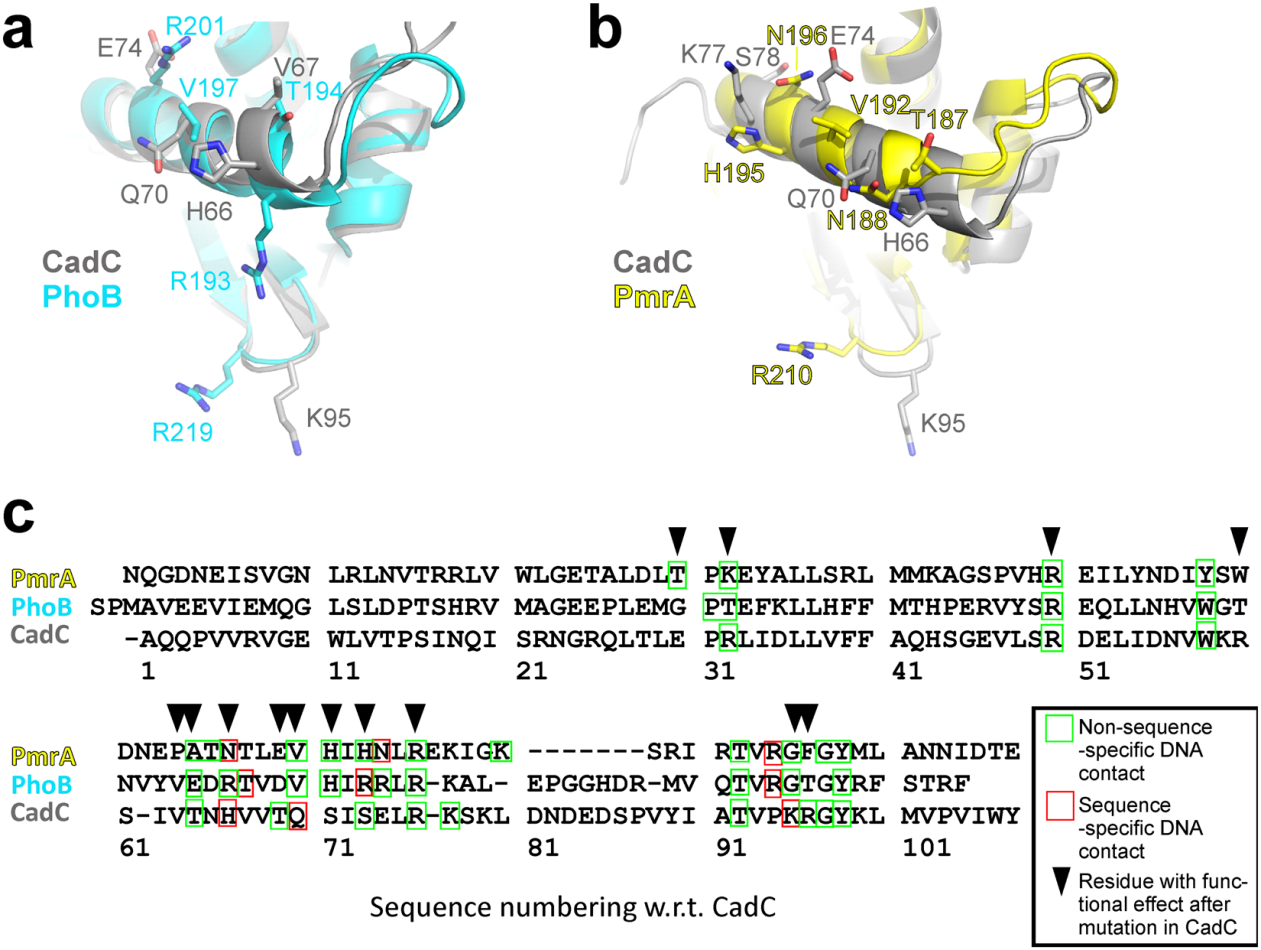
Structural comparison of DBD - DNA complexes. (**a**) Superposition of CadC_1-107_ and PhoB^16^ as taken from their complex structures. Shown is the recognition helix and the β-wing with analogous residues that are involved in specific interactions with DNA in at least one of the complex structures/model. Residues were selected from the structure-based alignment shown in (**c**). (**b**) The same as in (**a**) but with PmrA as taken from its complex structure with DNA^18^. (**c**) Structure-based sequence alignment of the CadC DBD and the DBDs from PhoB and PmrA with residues contacting DNA as indicated. Arrows above the sequence show residues with a functional impact after mutation in CadC (see Fig. 5).

We then compared the CadC-DNA complex model with two recently reported complex structures: the PhoB (RMSD 1.8 Å) and PmrA (RMSD 2.7 Å) DBDs (Fig. 8a and b). A structure-based sequence alignment of the three proteins shows that equivalent positions are used for contacting DNA (Fig. 8c) including the highly conserved residues Arg50, Trp58, Arg76, Thr92, and Tyr98. The three side chains that mediate sequence-specific contacts to the DNA are different in all three proteins (i.e. His66, Gln70 and Lys95 in CadC, see Fig. 8c), which reflects the distinct DNA sequence preferences. Interestingly, the number of residues that mediate sequence-specific interactions is identical in all complexes. However, the PmrA and PhoB DBDs show additional non-sequence-specific contacts involving residues in regions 30–32 and 71–74. Notably, the amino acid sequence for residues 71–74 is distinct from the corresponding regions in PhoB and PmrA and its importance is demonstrated by the observation that single amino acid replacements in this region in CadC also abolish its functional activity.

## Discussion

Bacterial effector domains in both one- and two-component signalling systems show diverse structural topologies^32^. The crystal structure of the CadC DBD resembles OmpR effector domains, but, to the best of our knowledge, represents the first structure of an effector domain from the ToxR-like transcriptional regulator family. Available structural and biochemical data demonstrate that dimerization of CadC is essential to mediate its functional activity in the cell in response to acid stress. Dimerization is mediated by the periplasmic domain^20^ and the transmembrane region^19^, while the linker is required for transducing the pH-dependent response of the periplasmic sensor into a structural rearrangement that facilitates dimerization of the cytoplasmic CadC DBD^21^. We have recently shown that the CadC DBD is not *per se* dimeric. This is confirmed by the monomeric state of the DBD in the crystal and in solution as evidenced by our NMR and SAXS data.

Recent work has shown that ToxR-like transcriptional regulators can bind DNA variably via their effector domains dominated by non-specific interactions, often activating numerous promoter regions^33, 34^. The CadC-DNA interface determined by our NMR chemical shift perturbation analysis is very similar to other complexes, e.g. PmrA and its target DNA^26^. Affinities between HTH DNA-binding domains and DNA can span several orders of magnitude depending on the specificity and stoichiometry of interactions^17, 18, 26, 34–38^. While two-component systems involve dimers of DBDs mediated by their receiver domains, CadC will only act as a dimer when anchored in the membrane. In line with our previous data^22^, the interaction of CadC_1-107_ with single motifs in Cad1 fragments is of comparably low (micromolar) affinity. However, the binding affinity is increased with the full-length Cad1 41-mer DNA and a Cad1 26-mer fragment that includes the two AT-rich recognition sites. The affinity of the wild type CadC DBD to its cognate Cad1 DNA (*K*_D_ = 380 nM as obtained by SPR) is in a typical range for two-site-binding^26, 34, 37^ although stronger interactions have been seen^38^. Notably, EMSAs and ITC revealed a 10-fold weaker *K*_D_ of CadC for the 41-mer and 26-mer DNAs than SPR. Comparing only EMSA experiments, the increase in affinity from 17-mer to 41-mer binding is only about 5-10-fold, which may reflect a lower off-rate of CadC from the 41-mer considering sliding of the protein on the longer DNA. The lower apparent *K*_D_ derived from the SPR data may be influenced by steady state conditions in the presence of immobilized DNA on a surface, which can overestimate binding affinities but consistently reports about relative affinities in our mutational studies (Fig. 5 and Supplementary Fig. 5). Nonetheless, we observed a comparable low affinity of CadC with Cad1 17-mer DNA by SPR (23 μM), NMR (67 μM) and EMSAs (60–67 μM). Altogether the data indicate that DNA affinity increases with the length of the DNA (due to lower off-rates and enhanced unspecific binding/sliding).

Dimerization of CadC enables the binding of two DBDs to the two Cad1 consensus target sites. Membrane-anchored CadC dimer will thus notably enhance the DNA-binding affinity which underlines an accepted hypothesis of transcriptional activation where multiple binding sites are the key to discrimination between transcription “on” and “off” states^39^. An affinity increase of CadC with full-length promoter DNA over individual sites was reported previously^22^ and is likely for other DBD-DNA complexes^17, 26^. Rearrangement of the transcription initiation complex may involve DNA bending by binding of multiple effector domains and was found for PhoB^16, 30, 40^. We propose a related mechanism for CadC: Our data show that the CadC DBD alone forms a 2:1 complex with 41-mer DNA increasing affinity. This affinity will be even more enhanced when a dimeric DBD is further stabilized by anchoring CadC in the membrane^21^.

For CadC in *V. vulnificus*, the presence of the leucine-responsive regulatory protein (Lrp) enhances the activation of the *cadBA* promoter through direct interaction of Lrp with DNA. DNA looping then enables protein-protein contacts between CadC and RNAP^41^. In a similar manner, the presence of multiple binding sites within the *cadBA* promoter in *E. coli* could promote cooperative DNA binding by CadC dimers in the membrane and thereby enhance the interaction with RNAP that, however, only takes full effect in stable CadC dimers.

Our structural and mutational analyses identified key residues in helix α3, the β-wing and the TL region to be important for interaction with the DNA and transcriptional initiation. Most surprisingly, charge inversion at residue Glu30 yields a constitutive complex with DNA and pH- and lysine-independent activity, which is expressed in its significantly higher DNA affinity. While CadC_1-159_-R96A has reasonable affinity for Cad1 *in vitro*, it fully fails to induce gene expression *in vivo*. Possibly, the loss in affinity is sufficient to overcome the threshold for transcription initiation by the RNAP. Not surprisingly, alanine replacement of the highly conserved residues Arg50 and Arg76 is not tolerated for DNA-binding or transcriptional activation. The ternary complex of CadC, DNA and RNAP likely requires a strict complex topology with key contacts present between enhancer and DNA.

Although structures of numerous effector domains are known and more than 60% of them have been shown to directly bind DNA^32^, high-resolution structural information of protein-DNA interactions has remained scarce^16–18^. We did not succeed in crystallizing CadC with Cad1 DNA and previous studies could not describe the target sequence in detail^22, 24^. Our two complex models provide insight into the DNA recognition by CadC and indicate a preference for AT-rich regions with a significant fraction of non-sequence-specific interactions compared to few sequence-specific ones. Predominantly non-specific interactions between transcriptional activators and DNA have been observed in other DBDs^16, 26^. The DNA consensus sequence 5′-T-T-A-x-x-x-x-T-3′ is present once in the quasi-palindromic Cad1 17-mer DNA, consistent with the formation of a 1:1 complex. However, a second consensus facilitates the formation of the 2:1 complex of CadC with Cad1 41-mer DNA as evidenced by the CadC model with the minimal Cad1 26-mer DNA that spans the two AT-rich regions, i.e. consensus sites. Therein, the two CadC DBDs form a dimeric head-to-head arrangement in line with the consensus site topology in Cad1. The sequence is also consistent with previous findings that CadC is able to replace the global repressor H-NS from AT-rich regions in the *cadBA* and *cadC* promoters^42, 43^. A sequence alignment of *cadBA* promoters shows that the AT-rich motif is also present in *V. cholerae* (Supplementary Fig. S6), consistent with a conserved DNA recognition by CadC in bacteria.

In summary, we propose that the presence of two DBDs by membrane-anchored CadC and a combination of AT-preference and non-sequence specific DNA contacts are important for cooperate DNA-binding and effective regulation of the *cadBA* promoter. In this respect it is noteworthy that cooperative DNA-binding has been proposed for ToxR-like proteins before^17, 26, 37, 44^. In addition, the architecture of the DNA-binding sites has a measurable influence on transcriptional control^45^. The presence of enhancer and promoter DNA guides RNAP to its point of action and thus provides spatiotemporal control of transcription initiation^40, 41^. Considering that the CadC dimer has a local cellular concentration at the membrane the regulation of *cadBA* expression may thus be less dependent on overall cellular CadC concentration and specificity-based affinity with target DNA. The formation of high-affinity complexes of CadC dimers with the promoter near the membrane may thus allow for a CadC-specific transcriptional regulation even though the recognition of the promoter does not involve high sequence specificity. Future structural and functional data will help to elucidate the molecular details and broaden our picture of CadC-DNA-RNAP interactions.

## Materials and Methods

### Cloning, production and purification of recombinant proteins

Expression vectors encoding the two CadC soluble variants CadC_1-107_ and CadC_1-159_ were generated by using standard procedures. We used the pETTrx1a vector provided by the Protein Expression and Purification Facility at the Helmholtz Zentrum München. All vectors encoded TEV protease recognition sites for subsequent proteolytic removal of the tags. The two proteins were produced as N-terminal His_6_-thioredoxin fusion proteins. A single fresh clone of *E. coli* BL21 DE3 cells was inoculated in lysogenic broth (LB)^46^ with 35 mg/l kanamycin and grown overnight. 1 ml was used to inoculate an expression culture. Cells were grown to an OD_600_ of 0.9 at 37 °C, induced with 0.5 mM IPTG and grown overnight at 20 °C before harvesting by centrifugation. Cells were resuspended in lysis buffer (50 mM Tris, 300 mM NaCl, 4 mM tris(2-carboxyethyl)phosphine (TCEP), 15 mM imidazole, 1 mg/ml lysozyme, 10 μg/ml DNaseI, and protease inhibitors, pH 8.0), incubated on ice for 30 min and sonicated. Cleared lysates were subjected to Ni^2+^-agarose beads. After intensive washing, beads were incubated with 500 µg/l TEV protease in lysis buffer under gentle shaking at room temperature for 3 h. Subsequently, the bead supernatant was collected, concentrated and gel-filtrated in 20 mM 2-[Bisamino]-2-1,3-propanediol (BisTris), 500 mM NaCl, pH 6.5. The respective protein monomer peak was pooled, and the salt concentration adjusted to 150 mM. CadC_1-159_ proteins were additionally purified via an anion exchange chromatography to remove proteolytic fragments. The protein was buffer exchanged to 20 mM Tris, 50 mM NaCl, and pH 8.0 and loaded onto a 1 ml MonoQ column. A gradient from 50–500 mM NaCl was applied over a volume of 10 ml. Fractions devoid of degradation were pooled and buffer exchanged to conditions as for CadC_1-107_. To obtain isotope-labelled proteins for NMR studies, cells were grown in M9 minimal medium supplemented with 0.5 g/l ^15^N ammonium chloride and 2 g/l unlabelled or [U-^13^C]-glucose. Amino acid replacements in CadC_1-159_ were introduced as described before^21^.

### DNAs

All DNAs were obtained as HPLC-purified single-stranded oligomers from MWGoperon (Ebersberg, Germany) or Sigma Aldrich (Deisenhofen, Germany). Individual strands were dissolved in water and complement molecules annealed by mixing stoichiometric ratios to a final concentration of 2 mM each and boiling them at 95 °C for 5 minutes. Samples were then cooled down to room temperature over 30 minutes and finally frozen at −80 °C. Sense strand sequences were: 5′-TTGTAAACATTAAATGTTTATCTTTTCATGATATCAACTTG-3′ (comprising base pairs -153 to -113 of the *cadBA* promoter, designated as Cad1 41-mer WT, see nucleotide exchanges for Cad1 ABmut in Supplementary Fig. S7), 5′-TAAACATTAAATGTTTA-3′ (−144 to −128 bp of the *cadBA* promoter, designated as Cad1 17-mer), 5′-GTAAACATTAAATG-3′ (Cad1A, 14-mer), 5′-GTTTATCTTTTCA-3′ (Cad1B, 13-mer), 5′-GTAAACATTAAATGTTTATCTTTTCA-3′ (Cad1AB, 26-mer), and 5′-TGATATCAACTTG-3′ (Cad1C, 13-mer).

### Crystallization, diffraction data collection and processing

Initial crystallization screening of CadC_1-107_ was set up at 292 K using 25 mg/ml of protein with a nanodrop dispenser in sitting-drop 96-well plates and commercial screens. Optimization was performed using the sitting-drop vapour-diffusion method at 292 K in 24-well plates. The crystals of CadC_1-107_ appeared after 14 days and required another 2 weeks to grow to the final size. The best diffracting crystals were obtained from 10 mM MES pH 6.5, 10 mM zinc sulphate, 27.5% (w/v) PEG550 MME. For the anomalous data collection, the native crystals were soaked with [Ta_6_Br_12_]^2+^ × 2 Br^−^ (Jena Bioscience). Native diffraction data was collected on the ID29 beamline (ESRF, Grenoble, France) using a PILATUS 6 M detector at a wavelength of 0.99987 Å. A [Ta_6_Br_12_]^2+^ × 2 Br^−^ derivatized crystal was used for the anomalous data collection at the tantalum absorption edge (1.25363 Å). Both data sets were collected at 100 K at the same beam line. All data sets were indexed and integrated using *XDS* ^47^ and scaled using *SCALA* ^48, 49^. Intensities were converted to structure factor amplitudes using the program *TRUNCATE* ^49, 50^. Supplementary Table S1 summarizes data collection and processing statistics for the two data sets.

### Structure determination and refinement

The structure of CadC_1-107_ was solved using the SAD protocol of Auto-Rickshaw, the EMBL-Hamburg automated crystal structure determination platform^51^. The input diffraction data were prepared and converted for use in Auto-Rickshaw using programs of the *CCP4* suite^49^. F_A_ values were calculated using the program *SHELXC* ^52^. Based on an initial analysis of the data, the maximum resolution for substructure determination and initial phase calculation was set to 2.6 Å. 15 heavy atom positions were located with the program *SHELXD* ^52^. The correct hand for the substructure was determined using the programs *ABS* ^53^ and *SHELXE* ^52^. The occupancy of all substructure atoms was refined using the program *MLPHARE* ^49^ and phases improved by density modification using the program *DM* ^49, 54^. The initial model was partially built using the program *ARP/wARP* ^55, 56^. Further model building and refinement using native data set (2.05 Å) was performed with *COOT* ^57^ and *REFMAC5* ^58^, respectively, using the maximum-likelihood target function including TLS parameters^59^. The final model is characterized by R and R_free_ factors of 18.3% and 22.3% (Supplementary Table S1). Stereochemical analysis of backbone dihedral angles in the final model using *PROCHECK* ^60^ revealed 95.3% of all CadC amino acids in the most favoured region and the remaining 4.7% in allowed regions of the Ramachandran plot. The refined structure and the corresponding structure factor amplitudes were deposited with the PDB under the accession code 5ju7. The crystal contains one molecule of CadC_1-107_ in the asymmetric unit. Well-defined electron density map corresponds to the region comprising Ala2 – Tyr107. For further analysis and validation, structure based alignments were performed with the DALI server^23^ using the complete crystal structure. We used 95 protein structures from a total of 56 PDB entries with a Z-score of or higher than 7.2 (i.e. half the maximum Z-score found for the best hit). RMSD values were between 1.6 A and 3.9 A. The 11 best-scored hits (i.e. with a Z-score above 13, but excluding redundant protein species in multiple entries) are listed in Supplementary Fig. S1.

### NMR spectroscopy

All NMR measurements of CadC_1-107_ and CadC_1-159_ were performed in 20 mM BisTris, 150 mM NaCl, pH 6.5 mixed with 10% (v/v) D_2_O. Backbone chemical shift assignments of both length versions and side chain assignments of CadC_1-107_ were recorded at protein concentrations of 1.2 mM (CadC_1-107_) and 600 μM (CadC_1-159_), respectively. HNCA, HNCACB, CBCAcoNH, HNCO, HNcaCO, HbHaNNH, HCCH-TOCSY and 3D ^15^N- and ^13^C-edited NOESY spectra^61^ were acquired at 298 K (RT) on BrukerAvance III spectrometers at field strengths corresponding to 600, 800 and 900 MHz proton Larmor frequency, equipped with TCI cryogenic probe heads. Amide ^15^N *R*_1_ and *R*_2_ relaxation data (e.g. to derive τ_C_ values) and steady-state heteronuclear {^1^H}-^15^N NOE experiments were performed as described^62^ at a temperature of 298 K and 600 MHz proton Larmor frequency. Spectra were processed with Topspin3.2 and analysed with CCPNMR Analysis^63^ and Sparky^64^.

Titrations of CadC_1-107_ or CadC_1-159_ with Cad1 DNA or fragments thereof were carried out at RT with protein concentrations of 100–300 μM in buffers as described above at molar ratios given in the respective figures. Chemical shifts were monitored using ^1^H-^15^N HSQC experiments and their perturbations (CSP) were calculated as described previously^65^. NMR relaxation data of the CadC_1-107_-Cad1 17-mer complex were measured at the final titration point. DNA imino proton resonances of Cad1 fragments alone and in complex with CadC_1-107_ were assigned using 2D imino NOESY spectra and SOFAST-HMQC experiments^66^. All 1D and 2D imino NOESY spectra were recorded using WATERGATE and water-flip-back pulses^67, 68^ at 600 MHz at a temperature range from 278 to 298 K and at 250 µM DNA concentrations. Spectra displayed in the respective imino proton 1D spectra were recorded at 283 K as unambiguous resonance assignments for DNA alone and in complex with CadC have only been obtained at this temperature. NOE cross peaks in 3D ^15^N- and ^13^C-edited NOESY spectra were automatically assigned with the program Cyana3^69^ and manually confirmed.

### CadC-DNA complex modelling

The complex of CadC_1-107_ with Cad1 17-mer DNA was modelled using the HADDOCK server^31^. We used the protein crystal structure as semi-flexible input. The Cad1-DNA was created with 3DNA^70^ using the DNA geometry from a PhoB-DNA complex structure^16^ and was otherwise kept rigid to avoid unfavourable strand melting at the DNA termini. However, with this we have automatically created a set of DNA restraints picked from the input structure (as listed in the HADDOCK dna_rna_restraints.def) including DNA base planarity (true for all), planarity in Watson-Crick base pairs (not restrained), phosphate backbone dihedral angles (restrained as from input), sugar pucker dihedral angles (DNA not restrained to A- or B-form) and Watson-Crick base pairing (true). Despite of limited flexibility we did not restrain the virtual C1’-C1’ bond length. Active nucleotides, i.e. those considered to be directly involved in CadC DBD interactions, were chosen based on the alignment of DNA promoter sequences with high ambiguity. I.e. multiple DNA nucleotides were considered for protein interaction (9, 11, 12, 13, 70, 71, 77, and 78) to allow the protein to move along the DNA double helix. The definition was also based on experimental chemical shift perturbations (CSPs) observed for the AT pairs, but not for the two GC pairs when adding CadC_1-107_ to Cad1 17-mer DNA (Supplementary Fig. S4). Ambiguous interaction restraints were derived from CSPs, i.e. the 10 most strongly affected amino acids used as active residues: Leu53, Val63, Val68, Thr69, Glu74, Arg76, Thr 92, Lys95, Tyr98, and Lys99. We also included the proximity of Glu30 to the DNA backbone. Water refinement by HADDOCK yielded 170 out of 200 structures in 12 clusters. The largest cluster (26 structures) which also showed least restraints violations and the lowest energy structure was chosen for further analysis. The first four structures in this cluster were among the eight lowest energy structures of all 200 models. The structural complex model was validated by mutational analysis and compared against all experimental CSPs. The complex of CadC_1-107_ with Cad1AB 26-mer DNA was modelled based on the DNA geometry from PDB entry 4kfc^38^ which was aligned to the final model of CadC_1-107_ with Cad1 17-mer DNA. While this arrangement was kept fixed the second CadC_1-107_ monomer was included with NMR-based restraints as mentioned above. The general settings for the HADDOCK run were identical with the CadC_1-107_-Cad1 17-mer complex modelling. HADDOCK yielded 9 clusters with 151 structures. The final cluster was ranked with the third best HADDOCK score and showed the lowest RMSD to the overall lowest-energy structure. It yielded the lowest overall enthalpy and contains the highest buried surface area and lowest AIR (ambiguous interaction restraints) violations. HADDOCK statistics for the two complexes are shown in Supplementary Table S3.

### SAXS measurements

SAXS measurements were performed on a Rigaku BIOSAXS1000 instrument with a HF007 microfocus generator equipped with a Cu-target at 40 kV and 30 mA. Transmissions were measured with a photodiode beam stop; q-calibration was made by a silver-behenate measurement. Measurements were carried out in multiple 900-second-frames, checked for beam damage and averaged. Circular averaging and background subtraction was done with the Rigaku *SAXSLab* software v 3.0.1r1. The distance distribution functions P(*r*) were calculated with GNOM, 3D models were generated using the DAMMIF software within the ATSAS package v 2.5.0-2^71^. Concentrations of samples were as follows: CadC_1-107_ (200 μM); CadC_1-107_ + Cad1 17-mer (each 200 μM); CadC_1-107_ + Cad1 41-mer (2:1: 267 μM + 134 μM); CadC_1-107_ + Cad1 41-mer (1:1: 160 μM + 162 μM); Cad1 41-mer alone (134 μM); and CadC_1-107_ + Cad1 26-mer (2:1: 200 μM + 100 μM). Buffers were identical to NMR conditions except for D_2_O. Molecular weights were calculated from Porod volumes. For complexes, the Porod volume of CadC was subtracted from the total volume and the protein weight calculated via its density. The remaining volume was processed using the density of DNA and finally both molecular weights combined again. CadC_1-107_-DNA *ab initio* shape models were created with dummy bead simulated annealing using the DAMMIF software within the ATSAS package v 2.5.0-2^71^. For both the 1:1 model of CadC with Cad1 17-mer and the 2:1 model of CadC with Cad1AB 26-mer DNA we manually fitted the experimentally obtained or HADDOCK-derived structures into the SAXS-derived shape.

### Static light scattering

Static Light Scattering (SLS) experiments of CadC_1-107_ were performed by connecting a Viscotek TDA 305 triple array detector to an Äkta Purifier equipped with an analytical size exclusion column at 4 °C. CadC samples in the presence of half-stoichiometric amounts of Cad1 41-mer or 26-mer DNA were run in gel filtration buffer on a GE Superdex200 10/300 column at 2 mg/ml protein concentration and a flow of 0.5 ml/min. The molecular masses of the samples were calculated from the experimentally determined refractive index and right-angle light scattering signals using Omnisec (Malvern Instruments). The SLS detector was calibrated with a 4 mg/ml BSA solution using 66.4 kDa for the BSA monomer and a dn/dc value of 0.185 ml/g for all protein samples.

### Surface Plasmon Resonance spectroscopy (Biacore)

SPR assays were performed in a Biacore T200 (GE Healthcare) using Xantec SAD500-L carboxymethyl dextran sensor chips pre-coated with streptavidin (XanTec Bioanalytics GmbH, Düsseldorf, Germany). All experiments were carried out at a constant temperature of 25 °C using 10 mM HEPES pH 7.4; 150 mM NaCl; 3 mM EDTA; 0.05% (v/v) detergent P20 as running buffer. Before immobilizing the DNA fragments, the chip was equilibrated by three injections using 1 M NaCl/50 mM NaOH at a flow rate of 10 µl/min. Then, 10 nM of the respective double-stranded biotinylated DNA fragment was injected using a contact time of 420 s and a flow rate of 10 µl/min. As a final wash step, 1 M NaCl/50 mM NaOH/50% (v/v) isopropanol was injected. Approximately 400 RU of Cad1-DNA fragment was captured onto flow cell 2. The surface of flow cell 1 was immobilized with similar amounts of *yhjX*-DNA and used to obtain blank sensorgrams for subtraction of bulk refractive index background. Interaction studies of CadC_1-159_-WT or the corresponding variants with the Cad1-DNA fragments were performed at 25 °C at a flow rate of 30 µl/min. The proteins were diluted in running buffer and passed over all flow cells in different concentrations (1 nM–100 nM) using a contact time of 180 s followed by a 300 s dissociation time. After each cycle, the surface was regenerated by injection of 2.5 M NaCl for 60 s at 30 µl/min flow rate followed by a second regeneration step with 0.5% (w/v) SDS for 60 s at 30 µl/min at 25 °C. Sensorgrams were recorded and analysed with the Biacore T200 Control software 2.0 and T200 Evaluation software 2.0 (GE Healthcare). The referenced sensorgrams were normalized to a baseline of zero. Peaks in the sensorgrams at the beginning and the end of the injection emerged from the runtime difference between the flow cells of each chip. For the calculation of *K*_D_ values, steady state affinity curves were used.

### ß-galactosidase assays to monitor *cadBA* expression

The effects of all CadC variants on *cadBA* promoter activation were determined using a β-galactosidase-based reporter gene assay. For this purpose the reporter strain *E. coli* EP314 (*cadC1*::Tn*10*, *cadA-lacZ* transcriptional fusion^28^) was transformed with plasmids encoding CadC or a variant of CadC (pET16b-based). Cells from an overnight culture aerobically grown in KE minimal medium (pH 7.6) supplemented with 0.2% (w/v) glucose^72^ were inoculated in fresh KE minimal medium containing 0.2% glucose (w/v) and buffered to pH 5.8 or pH 7.6 (100 mM phosphate buffer), and the cell density was adjusted to an OD_600_ of 0.05. Where indicated, lysine was added to a final concentration of 10 mM. Bacteria were grown to an OD_600_ of 0.3–0.5 under microaerobic conditions, and harvested by centrifugation. β-Galactosidase assays were performed as described previously^73^. Enzyme activity was determined from at least three independent replicates and is given in Miller Units^74^.

### EMSAs

Electrophoretic mobility shift assays (EMSAs) were performed by mixing 1.3 μg of Cad1 fragments or 1.9 μg of complete Cad1 with CadC_1-107_ in molar protein:DNA ratios of 0:1 to 10:1 in 20 mM BisTris and 150 mM NaCl, pH 6.5. Samples were incubated for 20 min at room temperature, mixed with native-PAGE loading buffer and loaded onto 15%- (fragments) or 12%- (Cad1 41-mer/26-mer) native polyacrylamide gels containing 5% (v/v) glycerol. Gels were run at conditions to avoid heating and maintain native complexes and finally stained with SYBRgold^®^. Quantification of complex formation was achieved by integration of bands with the Bio-Rad^®^ gel documentation system.

### ITC

Isothermal titration calorimetry (ITC) was performed with a MicroCal PEAQ-ITC device (Malvern, United Kingdom) in 20 mM BisTris and 150 mM NaCl, pH 6.5. In all experiments protein was titrated from a stock of 10-fold concentration excess to 40 µM Cad1 41-mer WT or ABmut or Cad1AB 26-mer DNA provided in the reaction cell. In a standard ITC run we used 19 injections of 2 µL with 120 seconds spacing at RT with a 750 rpm stirring speed. Raw data were analysed with the integrated software tool and heat production fitted to a one-site binding model. Cad1 41-mer DNA runs were performed in triplicate and the Cad1AB DNA in duplicate.

## Electronic supplementary material

Supplementary Information
