# Supplementary material for: Structure-function analysis of the DNA-binding domain of a transmembrane transcriptional activator
Source: Sci Rep. 2017 Apr 21;7:1051. doi: 10.1038/s41598-017-01031-9 (PMC5430869; doi:10.1038/s41598-017-01031-9)
Supplement: Supplementary file 1 — Supplementary Information [file 41598_2017_1031_MOESM1_ESM.pdf]

Supplementary Information on

Structure-function analysis of the DNA-binding domain of a transmembrane transcriptional activator

Andreas Schlundt[1,2], Sophie Buchner[3], Robert Janowski[4], Thomas Heydenreich[1], Ralf Heermann[3], Jürgen Lassak[3], Arie Geerlof[2], Ralf Stehle[1,2], Dierk Niessing[4,5], Kirsten Jung[3]*, Michael Sattler[1,2,*]

[1]Munich Center for Integrated Protein Science (CiPSM) at the Department of Chemistry, Technische Universität München, 85748 Garching, Germany

[2]Institute of Structural Biology, Helmholtz Zentrum München, 85764 Neuherberg, Germany

[3]Munich Center for Integrated Protein Science (CiPSM) at the Department of Microbiology, Ludwig-Maximilians-Universität München, 82152 Martinsried, Germany

[4]Group Intracellular Transport and RNA Biology at the Institute of Structural Biology, Helmholtz Zentrum München, 85764 Neuherberg, Germany

[5]Department of Cell Biology at the Biomedical Center, Ludwig-Maximilians-Universität München, 82152 Martinsried, Germany

* To whom correspondence should be addressed. Tel: +49 (89) 289 13418; Fax: +49 (89) 289 13869; Email: sattler@helmholtz-muenchen.de.
Correspondence may also be addressed to Kirsten Jung: Tel: +49 89 / 2180-74500; Fax: +49 89 / 2180-74520; E-mail: Kirsten.Jung@lrz.uni-muenchen.de

Supplementary Table S1. Data collection and refinement statistics for $CadC_{1\text{-}107}$.

	$[Ta_6Br_{12}]^{2+}$x 2 Br^- derivative	Native
**Data collection**		
Space group	$P6_422$	$P6_422$
Cell dimensions		
a, *b*, *c* (Å)	103.58, 103.58, 43.91	104.14, 104.14, 44.00
α, β, γ (°)	90, 90, 120	90, 90, 120
Resolution (Å)	50–2.3 (2.36–2.30)*	50–2.05 (2.10–2.05)
R_{merge}	6.5 (84.4)	3.8 (59.3)
$I / \sigma I$	35.2 (4.8)	35.8 (4.1)
Completeness (%)	99.8 (99.3)	99.6 (99.9)
Redundancy	36.9 (34.8)	13.6 (14.6)
**Refinement**		
Resolution (Å)		2.05
No. reflections		8,762
R_{work} / R_{free}		18.3 / 22.3
No. atoms		
Protein		870
Ions		5
Water		28
B-factor overall		48.9
R.m.s. deviations		
Bond lengths (Å)		0.02
Bond angles (°)		2.17

For each data set, only one crystal has been used. *Values in parentheses are for highest-resolution shell.

Supplementary Table S2. Summary of all SAXS measurements made with $CadC_{1-107}$ in complex with Cad1 17-mer or 41-mer DNAs.

	Rg [Å]	D_{max} [Å]	V_p [$Å^3$]	MW [kD]	Theoretical MW [kD]
$CadC_{1-107}$	13.8 ± 0.2	45	14842	12.3	12
$CadC_{1-107}$+ DNA17-mer (Cad1)	17.8 ± 0.1	62	21513	23.6*	22
$CadC_{1-107}$+ DNA41-mer (Cad1) 2:1	26.5 ± 0.2	93	47003	54.1*	48
$CadC_{1-107}$+ DNA41-mer (Cad1) 1:1	26.9 ± 0.2	95	38516	--	36
DNA41-mer (Cad1)	26.0 ± 0.3	93	17729	30.1*	24
$CadC_{1-107}$+ DNA26-mer (Cad1) 2:1	25.5 ± 0.2	88	43928	48.6*	41

* Note that the higher molecular weights in presence of DNA when compared to the theoretical ones are caused by the DNA hydration shell.

Supplementary Table S3. Statistics from HADDOCK run of $CadC_{1-107}$ in complex with Cad1 17-mer and Cad1AB 26-mer DNAs.

	Cad1 17-mer		Cad1AB 26-mer
	cluster (26 structures)	best 4*	cluster (4 structures)$
R.m.s. deviations (Å)	2.3 ± 0.8	1.2 ± 0.8	1.0 ± 0.6
HADDOCK score	-55 ± 16	-78 ± 2	-113 ± 9
Restraints violation			
Distance (Å)	1.92 ± 0.87	1.25 ± 0.43	2.25 ± 0.83
Dihedral (°)	0.54 ± 0.50	0.5 ± 0.5	0.00 ± 0.00
buried surface area (Å^2)	1165 ± 162	1386 ± 32	1665 ± 48

***Protein only R.m.s. deviations (Å) (2-107): Heavy atom: 1.15 ± 0.03; Backbone: 0.62 ± 0.03**
$Protein only R.m.s. deviations (Å) (2-107): Heavy atom: 1.19 ± 0.17; Backbone: 0.76 ± 0.10

Supplementary Fig. S1:

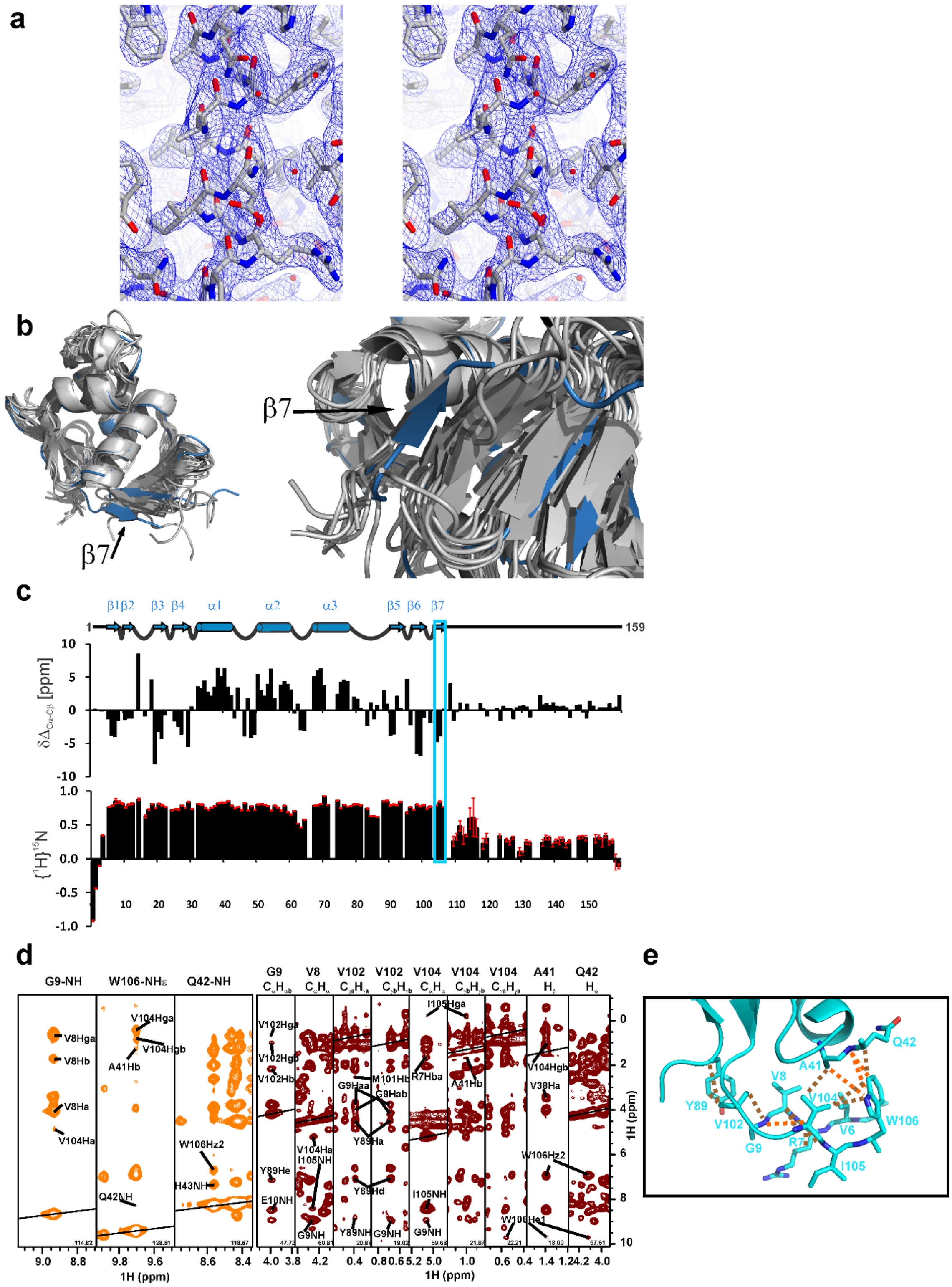

Supplementary Fig. S1. Analysis of the crystal structure of CadC$_{1\text{-}107}$ and validation in solution. (a) Stereo view of 2Fo-Fc electron density map displayed for helix α1 (Pro31-His43) of the CadC$_{1\text{-}107}$ crystal structure shown in main text Fig. 1 (map contoured at 1σ). The picture was prepared in Cuemol. **(b)** Left, overlay of CadC$_{1\text{-}107}$ (dark blue) with the 11 most similar structures found by DALI. The aligned structures are shown in grey and all comprise the essentially identical core fold with recognition helix α3 and β-loop-β wing motif. The right panel shows a magnification of the seventh β-strand in CadC$_{1\text{-}107}$ that is missing in any of the other structures (see arrows). The displayed structures are full or parts of PDB entries (DALI Z-score/RMSD in Å): 2pmu (14.4/2.1)[1], 2zxj (14.4/1.9), 3r0j (14.3/1.8)[2], 4ixa (14.0/1.8), 1qqi (13.9/1.9)[3], 2hwv (13.8/2.0)[4], 3rjp (13.6/1.6), 1gxp (13.5/1.8)[5], 2d1v (13.4/2.2)[6], 2gwr (13.2/2.5)[7], and 4kfc (13.2/2.6)[8]. **(c)** Top, secondary structure elements of CadC$_{1\text{-}159}$ as adapted from Buchner *et al.*, 2015 and in line with the crystal structure of the DBD (amino acids 1-107). Middle, secondary chemical shift plot for CadC$_{1\text{-}159}$. Gaps indicate prolines, glycines or residues with incomplete assignments for Cβ. Residues 105 to 107 are clearly structured and their negative secondary chemical shifts indicate a short β-strand (see boxed residues). Bottom, heteronuclear NOE data of CadC$_{1\text{-}159}$ as taken from Buchner *et al.*, 2015[9]. **(d)** 3D-^{1}H-^{15}N- (left) and 3D-^{1}H-^{13}C- (right) NOESY strips showing NOEs between CadC$_{1\text{-}107}$ β-strands 1 and 7. The respective root resonance is given above the strip. **(e)** The crystal structure excerpt is shown to visualize the selected NOEs with the colour code as for peaks in B.

Supplementary Fig. S2:

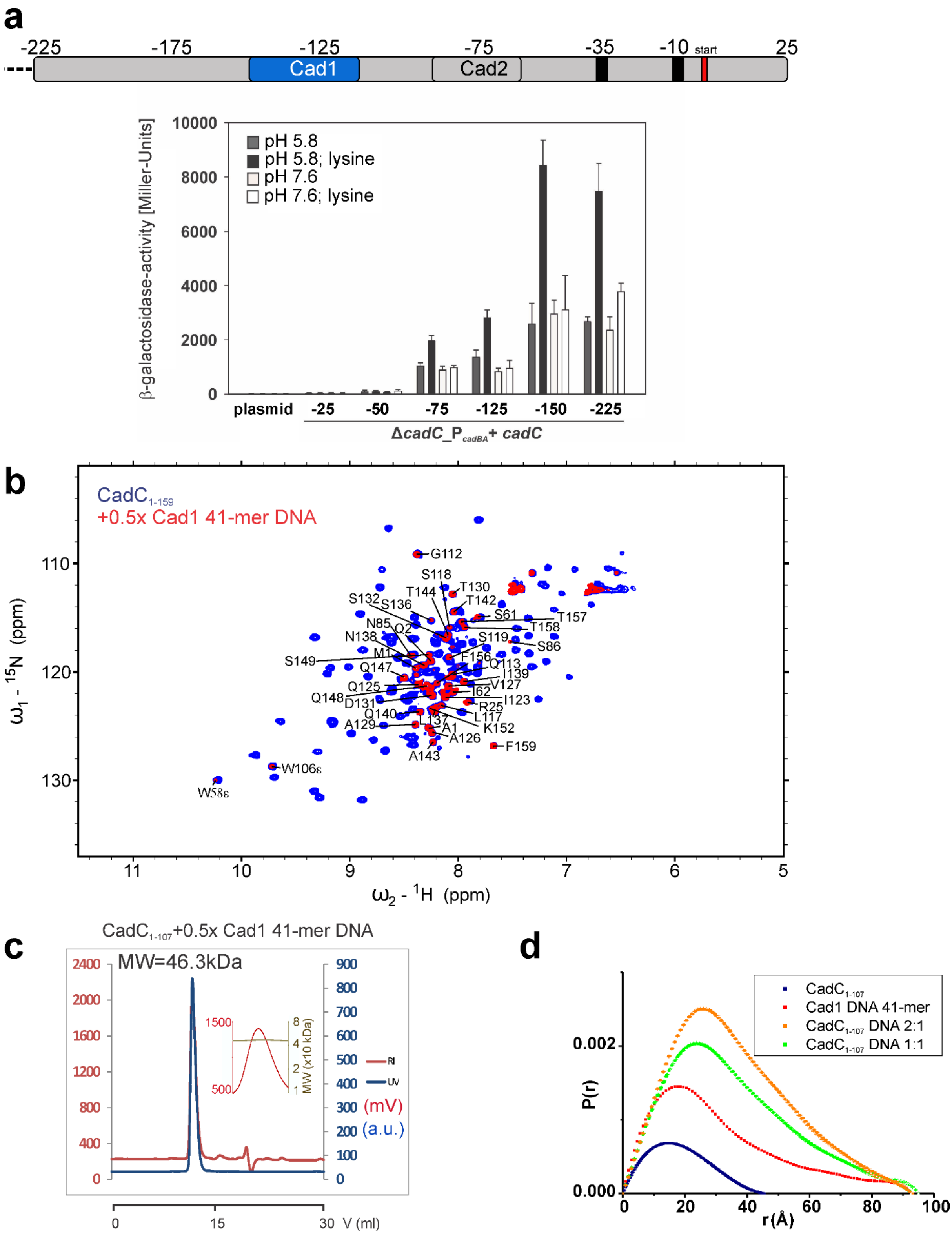

Supplementary Fig. S2. Analysis of the Cad1 41mer in complex with the CadC DBD. (a) Top, scheme of the full *cadBA* promoter region between -175 bp and 25 bp relative to the transcription start with CadC binding sites Cad1 and Cad2 and the RNA polymerase binding sites at -35 bp and -10 bp. Bottom, reporter assay showing the β-galactosidase activity in indicated conditions in dependence of the *cadBA* promoter composition. Numbers indicate the 5'-end as upstream of the transcription start. Errors are standard deviations from three independent experiments. **(b)** HSQC overlay of $CadC_{1-159}$ alone or in complex with 0.5-fold Cad1 41-mer DNA. Selected peaks that are not line-broadened after DNA addition are shown with their assignments. **(c)** Static light scattering coupled to analytical gel filtration. The chromatogram shows $CadC_{1-107}$ in complex with half-stoichiometric Cad1 41-mer DNA. The inset shows the derivation of the molecular weight (MW, right axis, binary logarithmic scale). The theoretical MW is 48 kDa. **(d)** Particle size distributions from SAXS experiments for $CadC_{1-107}$, Cad1 41-mer DNA and complexes as given in the legend. Plots are normalized to concentrations. SAXS-derived parameters are summarized in Supplementary Table 3 and show that that determined Porod volumes and molecular weights of DNA and protein species add up in the complex samples.

Supplementary Fig. S3:

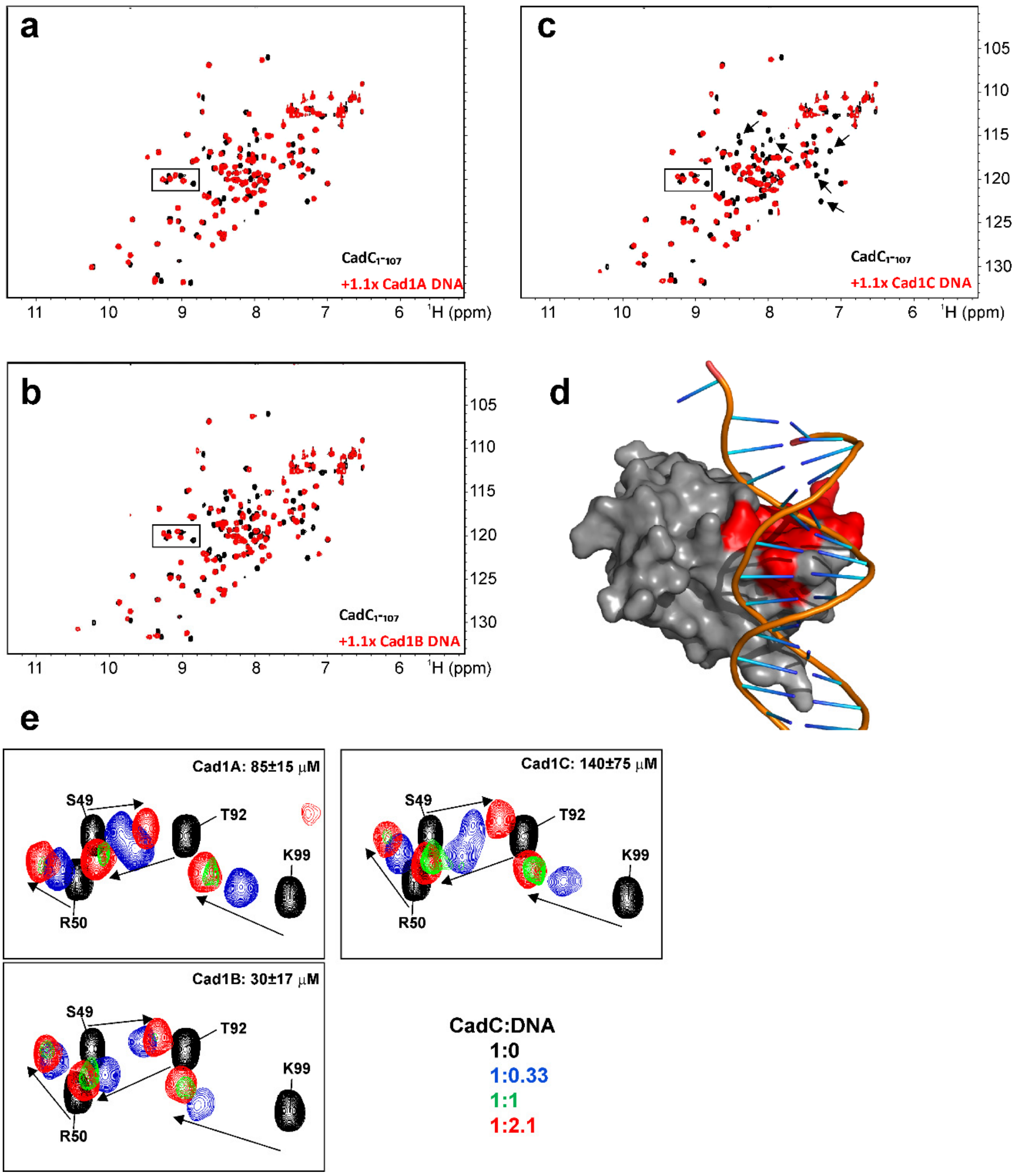

Supplementary Fig. S3. Protein NMR analysis of CadC$_{1\text{-}107}$ in complex with Cad1A, B and C. (a)-(c) HSQC overlays showing either CadC$_{1\text{-}107}$ alone (black) or in presence of Cad1A **(a)**, Cad1B **(b)** or Cad1C **(c)**. Arrows in panel C indicate line-broadening of peaks beyond detectability after addition of Cad1C DNA. **(d)** Visualization of line-broadened peaks in C on the surface of CadC in a complex model with *pho* DNA. **(e)** Insets from boxes in panels **(a)-(c)** showing four titration points (out of six in total) as indicated and assignments of selected CadC resonances. Dissociation constants from fitting of CSPs are given as means of at least 10 peaks and the standard deviation is given.

Supplementary Fig. S4:

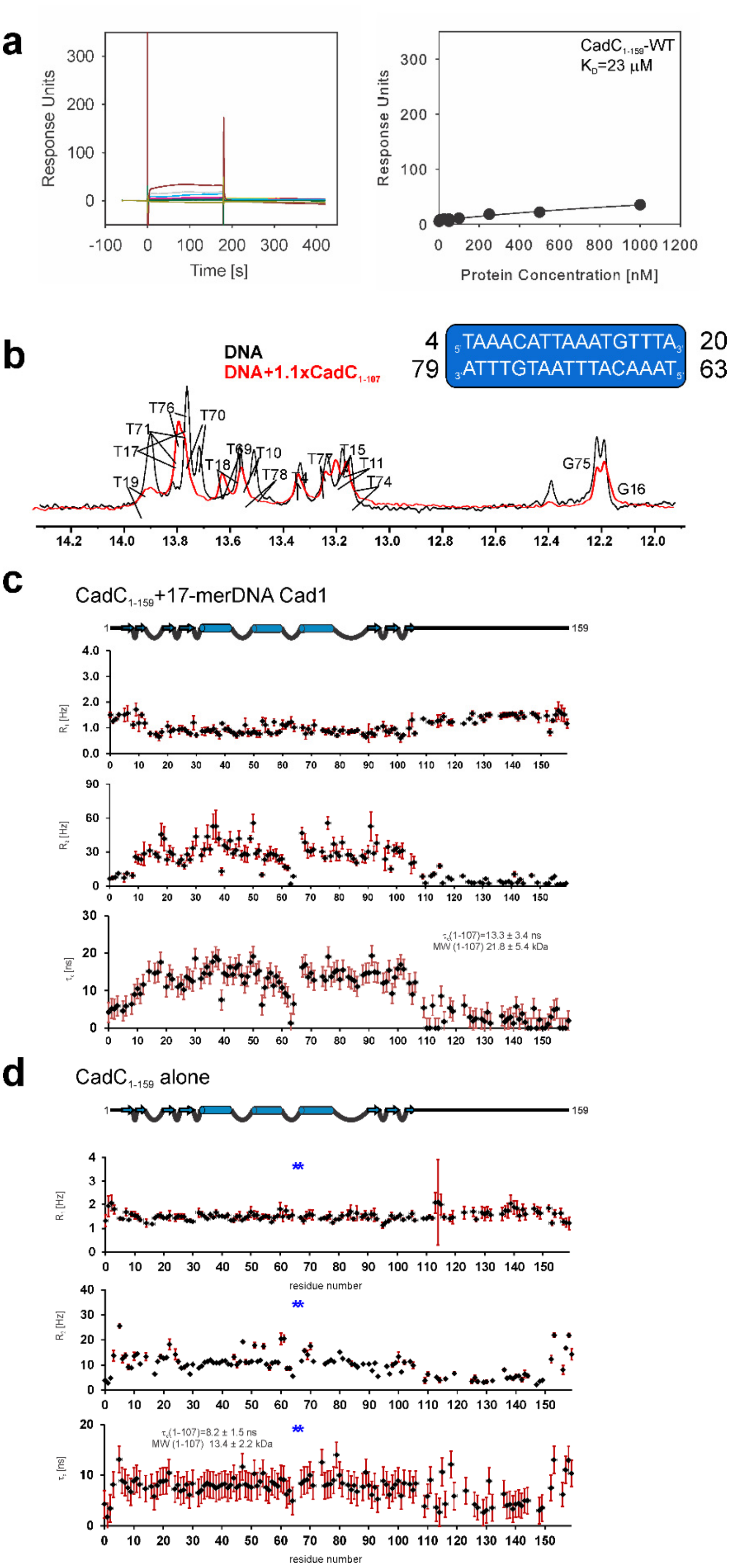

Supplementary Fig. S4. SPR and NMR analysis of the Cad1 17mer in complex with the CadC DBD. (a) SPR Sensogram and binding curve for CadC DBD when titrated to Cad1 17-mer DNA. Overall steady-state affinity was fitted to derive the binding constant. For colour code see main text Fig. 5. **(b)** 1D imino proton spectra of Cad1 17-mer DNA either alone (black) or in complex with one equivalent of $CadC_{1\text{-}107}$ (red). A-T pairs and G-C pairs are indicated by assignment of T and G bases, respectively. **(c)** NMR ^{15}N relaxation rates R_1 and R_2 for $CadC_{1\text{-}159}$ in complex with the Cad1 17-mer DNA. The total correlation time τ_c as a measure of the ratio from both rates is shown below, and the MW for the DBD (structured residues 1-107) was calculated from the averaged tumbling time of all structured residues. The theoretical MW for this complex is 22 kDa. **(d)** The same as in the top panel, but for unbound $CadC_{1\text{-}159}$ alone. The theoretical MW of the CadC stretch comprising the DBD (structured residues 1-107) is 12.6 kDa. Data in the bottom panel have been published before and are adapted from Buchner *et al.*, 2015[9]. Blue asterisks indicate the position of residues Asn65 and His66, for which no assignment was obtained due to line-broadening. Error bars in C and D are derived from the fitting uncertainty for R_1 and R_2 or are based on error propagation for τ_c. Expected values for τ_c are approximately 7.5 ns and 13.5 ns for $CadC_{1\text{-}159}$ alone and in complex with Cad1 17-mer DNA (based on the structured region of residues 1-107) according to the correlation of molecular weight and surface-accessible area with correlation times at 298 K when measured at room temperature [10,11]. To this end, extrapolation of τ_c values from experimental data obtained under identical conditions as for CadC has been performed with input data kindly provided by the NorthEast Structural Genomics consortium (James Aramini, University of Buffalo).

Supplementary Fig. S5:

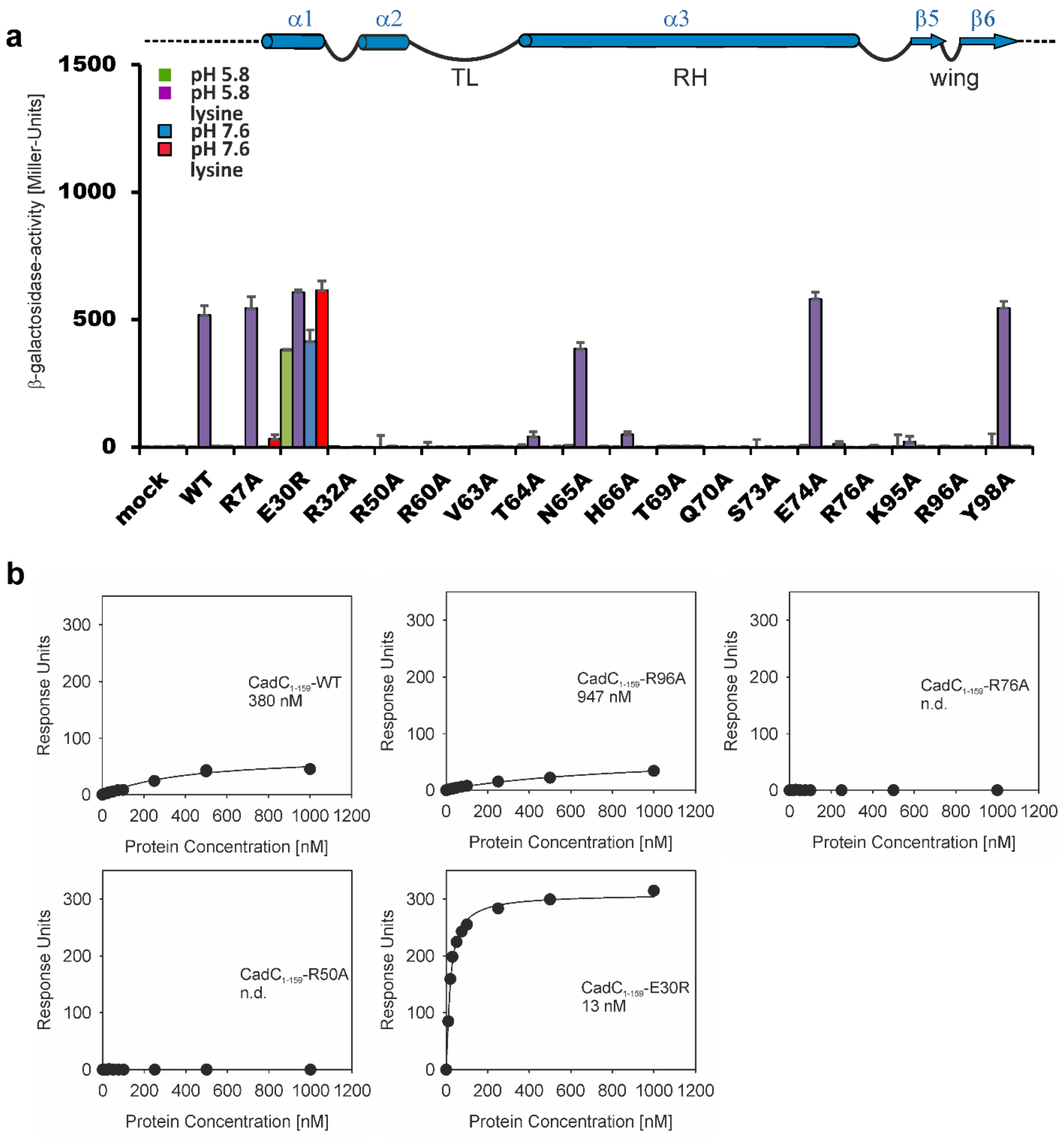

Supplementary Fig. S5. Influence of amino acid substitutions within CadC on the expression of the *cadBA* operon. (a) Quantification of *cadBA*-transcription in *E. coli* EP314 using the reporter gene *lacZ*. Cells were grown at the four different experimental conditions indicated, and the β-galactosidase-activity determined. The experiment was performed in triplicate, and, and error bars indicate standard deviations from the means. See also main text Fig. 5a and b. **(b)** Binding curves for CadC DBD when titrated to Cad1 41mer DNA derived from SPR raw data in main text Fig. 5c. Overall steady-state affinity was fitted to derive binding constants.

Supplementary Fig. S6:

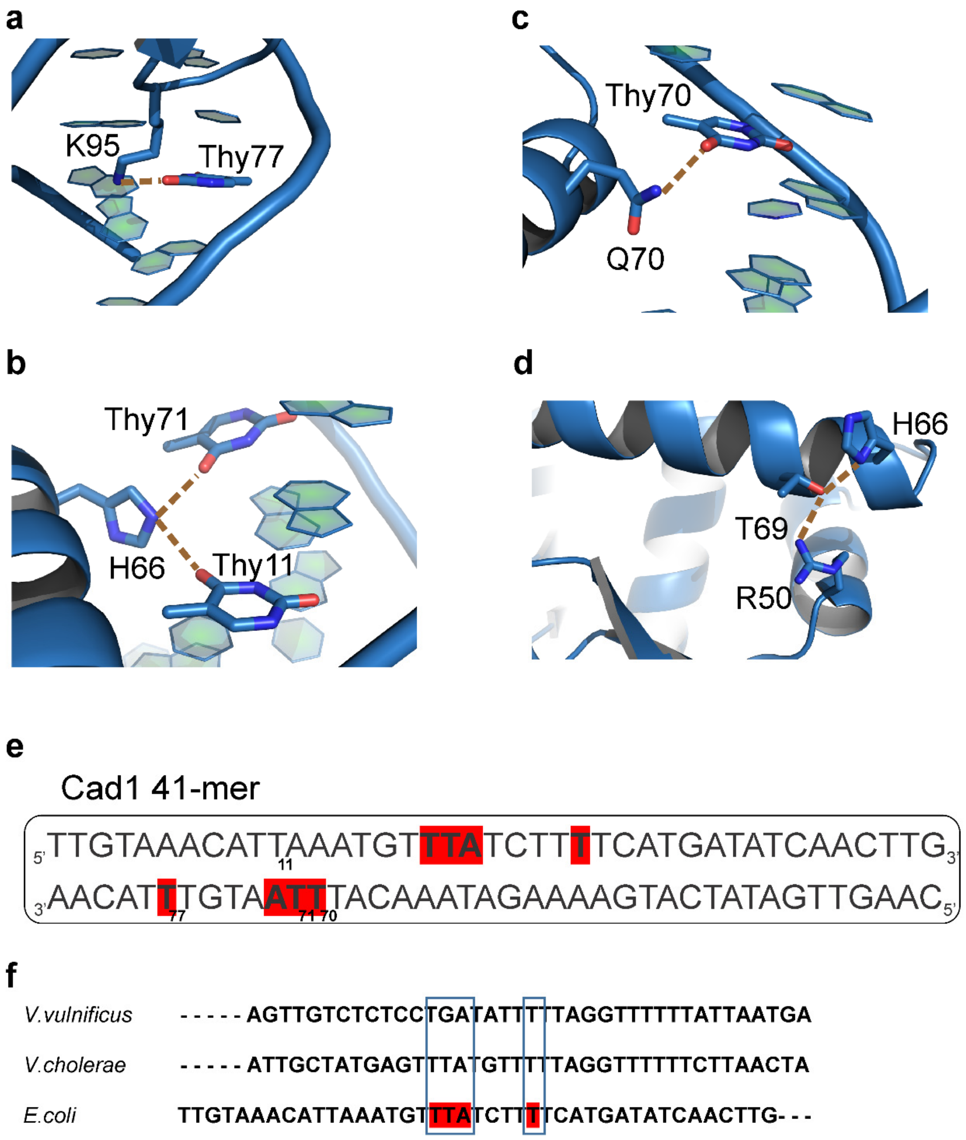

Supplementary Fig. S6. Detailed analysis of the HADDOCK-based CadC$_{1\text{-}107}$-Cad1 DNA complex model. (a)-(c) Zoomed-in structural views showing H-bond interactions between 17-mer Cad1 DNA and CadC side chains of residues Lys95, His66 and Gln70, respectively. The complex represents the lowest-energy HADDOCK model shown in main text Fig. 6. Only relevant amino acid side chains and bases are shown as sticks. Neighbouring bases are indicated. **(d)** Zoomed-in view showing intra-protein interactions for Thr69. The residue was found to be crucial for CadC-DNA interactions according to main text Fig. 5a. **(e)** The two potential binding sites of the CadC effector domain in Cad1 41-mer DNA as suggested by a putative consensus based on the specific protein-DNA contacts in **(a)-(c)**. Bases in red represent the consensus sequence. **(f)** Sequence alignment of the Cad1 41mer with the identified CadC binding sites in PcadBA of Vibrio *vulnificus* and Vibrio *cholerae*. Red letters indicate one potential CadC binding site in the E.coli Cad1 region and boxes refer to analogous nucleotides.

Supplementary Fig. S7:

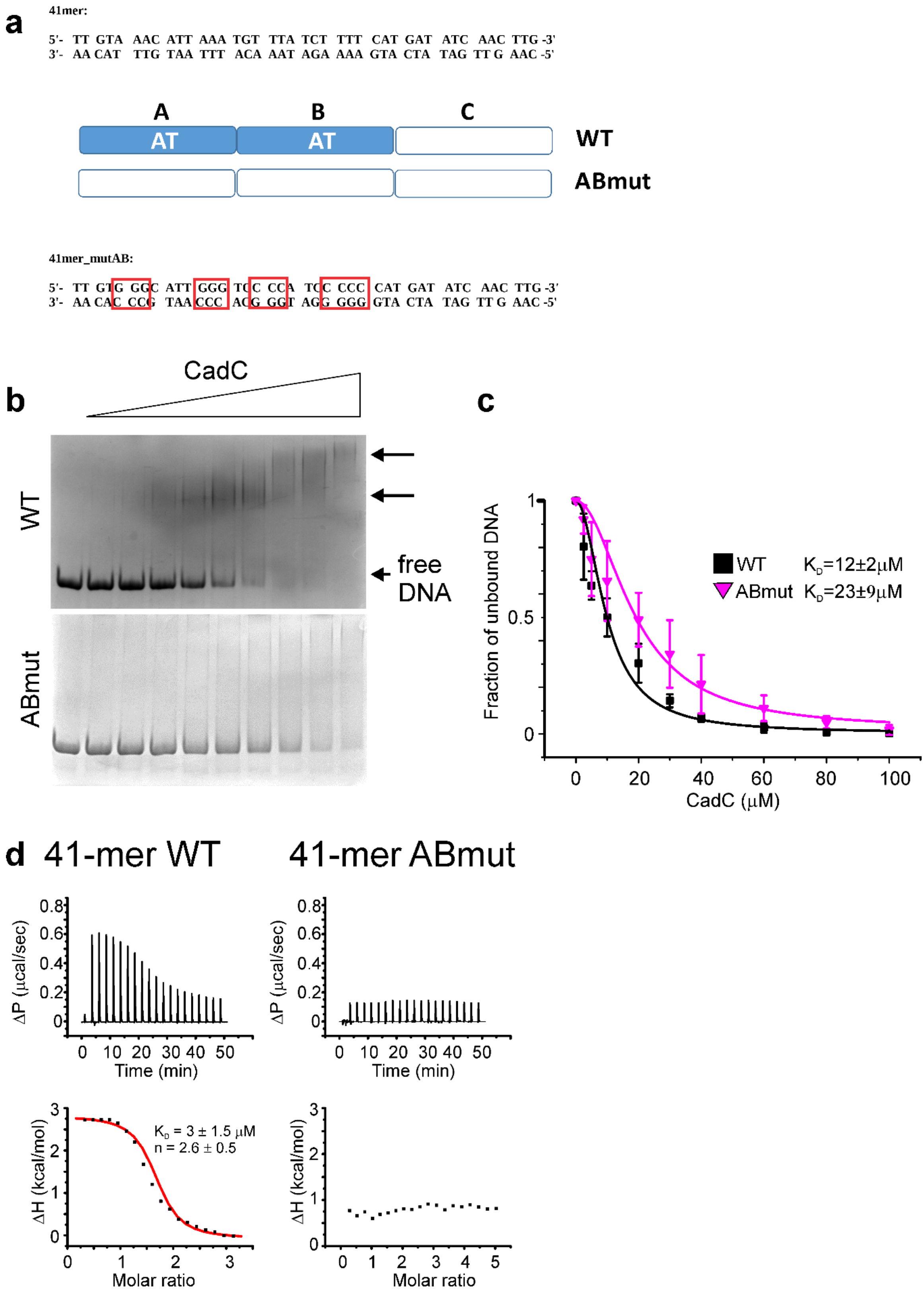

Supplementary Fig. S7. Analysis of AT-rich patches in Cad1. (a) Scheme of Cad1 41-mer DNA region and the two variants used for B. The three fragments indicated here resemble fragments Cad1, B and C in main text Fig. 2 and 3. Removing AT-rich patches was performed by six- and sevenfold exchange of ATs for CG for fragments A and B, respectively (see sequences. **(b)** Shown are representative gels of Cad1 41-mer DNAs as indicated and annotated in panel **(a)** when titrated up to tenfold excess of $CadC_{1-107}$. The band of free DNA is labelled and was used for quantification of the complex formation. Arrows indicate complexes found in the titration of WT DNA with CadC DBD. **(c)** Quantification of EMSA experiments of $CadC_{1-107}$ in B with either of the DNAs in **(a)**. Experiments were performed in triplicates with errors giving the standard deviations of experimental means. Dissociation constants are given in the diagram. (**d**) ITC measurements of Cad1 41-mer WT or ABmut DNA when titrated with $CadC_{1-107}$. Affinity and stoichiometry are given as mean±standard deviation from a triplicate or duplicate experiment for WT and ABmut, respectively.

Supplementary Fig. S8:

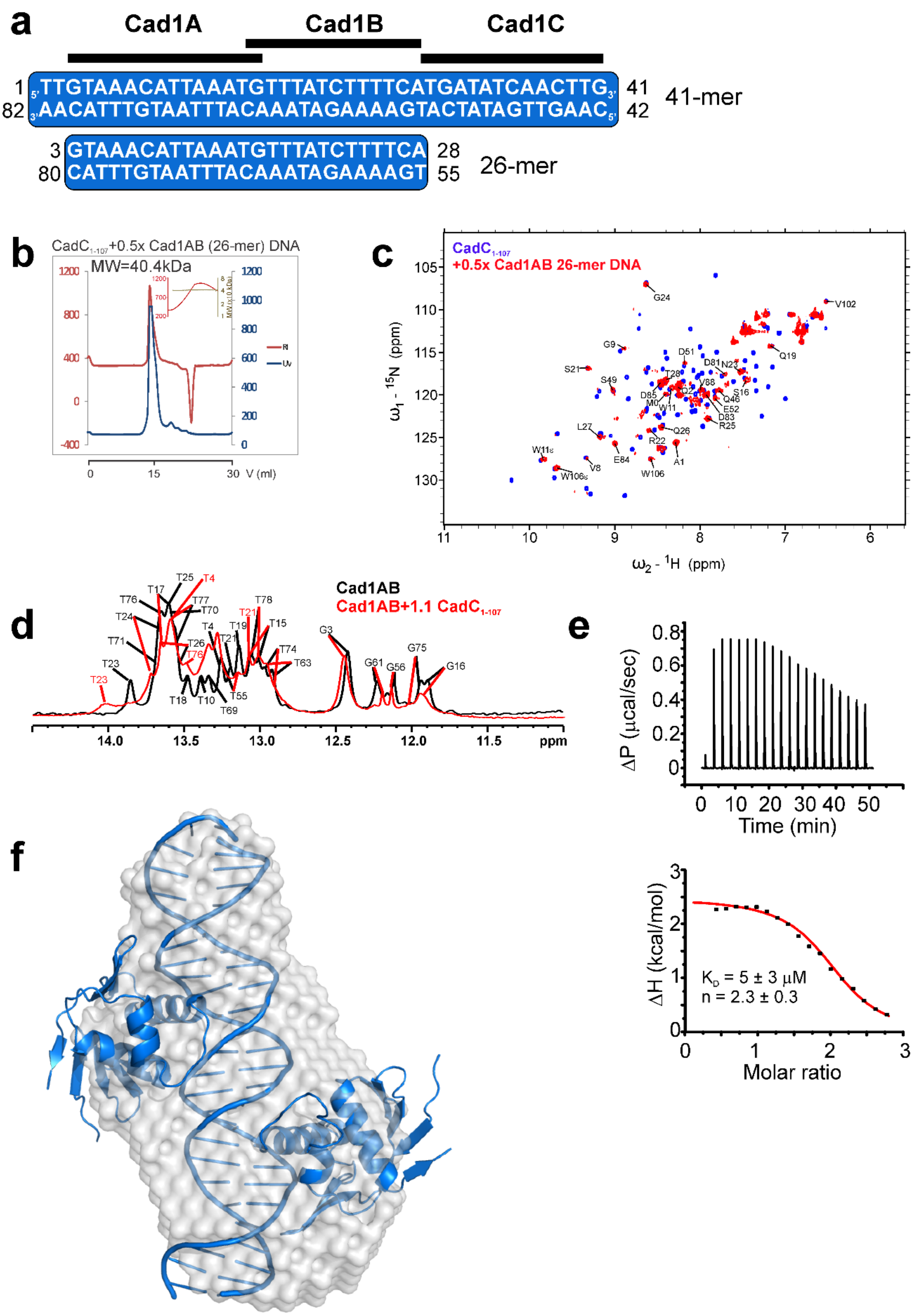

Supplementary Fig. S8. Analysis of Cad1AB 26-mer DNA. (a) Overview of the full-length Cad1 promoter region (41-mer) and the internal 26-mer Cad1AB fragment. **(b)** Static light scattering coupled to analytical gel filtration. The chromatogram shows $CadC_{1\text{-}107}$ in complex with half-stoichiometric Cad1AB 26-mer DNA. The inset shows the derivation of the molecular weight (MW, right axis). The theoretical MW is 40.8 kDa. **(c)** HSQC overlay of $CadC_{1\text{-}107}$ alone or in complex with 0.5-fold Cad1AB 26-mer DNA. Selected peaks that are not line-broadened after DNA addition are shown with their assignments. **(d)** 1D imino proton spectra of Cad1AB 26-mer DNA either alone (black) or in complex with one equivalent of $CadC_{1\text{-}107}$ (red). All obtainable assignments are shown. **(e)** ITC measurements of Cad1AB 26-mer DNA when titrated with $CadC_{1\text{-}107}$. Affinity and stoichiometry are given as mean±standard deviation from a duplicate experiment. **(f)** Model of $CadC_{1\text{-}107}$ bound to 26-mer Cad1AB DNA with a 2:1 stoichiometry as derived from SAXS. The ab initio complex model obtained from DAMMIF is shown in grey. The 26-mer DNA in complex with two molecules of $CadC_{1\text{-}107}$ is shown in blue.

Supplementary references

1. Wang, S., Engohang-Ndong, J. & Smith, I. Structure of the DNA-binding domain of the response regulator PhoP from Mycobacterium tuberculosis. *Biochemistry* **46**, 14751-61 (2007).
2. Menon, S. & Wang, S. Structure of the response regulator PhoP from Mycobacterium tuberculosis reveals a dimer through the receiver domain. *Biochemistry* **50**, 5948-57 (2011).
3. Okamura, H., Hanaoka, S., Nagadoi, A., Makino, K. & Nishimura, Y. Structural comparison of the PhoB and OmpR DNA-binding/transactivation domains and the arrangement of PhoB molecules on the phosphate box. *J Mol Biol* **295**, 1225-36 (2000).
4. Trinh, C.H., Liu, Y., Phillips, S.E. & Phillips-Jones, M.K. Structure of the response regulator VicR DNA-binding domain. *Acta Crystallogr D Biol Crystallogr* **63**, 266-9 (2007).
5. Blanco, A.G., Sola, M., Gomis-Ruth, F.X. & Coll, M. Tandem DNA recognition by PhoB, a two-component signal transduction transcriptional activator. *Structure* **10**, 701-13 (2002).
6. Okajima, T. et al. Response regulator YycF essential for bacterial growth: X-ray crystal structure of the DNA-binding domain and its PhoB-like DNA recognition motif. *FEBS Lett* **582**, 3434-8 (2008).
7. Friedland, N. et al. Domain orientation in the inactive response regulator Mycobacterium tuberculosis MtrA provides a barrier to activation. *Biochemistry* **46**, 6733-43 (2007).
8. Narayanan, A., Kumar, S., Evrard, A.N., Paul, L.N. & Yernool, D.A. An asymmetric heterodomain interface stabilizes a response regulator-DNA complex. *Nat Commun* **5**, 3282 (2014).
9. Buchner, S., Schlundt, A., Lassak, J., Sattler, M. & Jung, K. Structural and Functional Analysis of the Signal-Transducing Linker in the pH-Responsive One-Component System CadC of Escherichia coli. *J Mol Biol* **427**, 2548-61 (2015).
10. Krishnan, V.V. & Cosman, M. An empirical relationship between rotational correlation time and solvent accessible surface area. *J Biomol NMR* **12**, 177-82 (1998).
11. Garcia de la Torre, J., Huertas, M.L. & Carrasco, B. HYDRONMR: prediction of NMR relaxation of globular proteins from atomic-level structures and hydrodynamic calculations. *J Magn Reson* **147**, 138-46 (2000).
